# Efficacy and Safety of Colistin versus Tigecycline for Multi-Drug-Resistant and Extensively Drug-Resistant Gram-Negative Pathogens—A Meta-Analysis

**DOI:** 10.3390/antibiotics11111630

**Published:** 2022-11-15

**Authors:** Dina Abushanab, Ziad G. Nasr, Daoud Al-Badriyeh

**Affiliations:** 1 Department of Pharmacy, Hamad Medical Corporation, Doha 3050, Qatar; 2 College of Pharmacy, QU Health, Qatar University, Doha 2713, Qatar

**Keywords:** colistin, extensively drug-resistant, infection, multi-drug resistant, tigecycline

## Abstract

Background: We intended to compare the efficacy and safety outcomes of colistin versus tigecycline as monotherapy or combination therapy against multi-drug resistant (MDR) and extensively drug-resistant (XDR) pathogens. Methods: A search was conducted in PubMed, Cochrane CENTRAL, EMBASE, and in the grey literature (i.e., ClinicalTrials.gov and Google Scholar) up to May 2021. Outcomes were clinical response, mortality, infection recurrence, and renal and hepatic toxicity. We pooled odd ratios (OR) using heterogeneity-guided random or fixed models at a statistical significance of *p* < 0.05. Results: Fourteen observational studies involving 1163 MDR/XDR pathogens, receiving tigecycline versus colistin monotherapy or combination, were included. Base-case analyses revealed insignificant differences in the clinical response, reinfection, and hepatic impairment. The 30-day mortality was significantly relatively reduced with tigecycline monotherapy (OR = 0.35, 95% CI 0.16–0.75, *p* = 0.007). The colistin monotherapy significantly relatively reduced in-hospital mortality (OR = 2.27, 95%CI 1.24–4.16, *p* = 0.008). Renal impairment rates were lower with tigecycline monotherapy or in combination, and were lower with monotherapy versus colistin-tigecycline combination. Low-risk of bias and moderate/high evidence quality were associated with all studies. Conclusions: Within the limitations of this study, it can be concluded that there were no statistically significant differences in main efficacy outcomes between colistin and tigecycline monotherapies or combinations against MDR/XDR infections, except for lower rates of 30-day mortality with tigecycline and in-hospital mortality with colistin. Tigecycline was associated with favourable renal toxicity outcomes.

## 1. Introduction

Multi-drug resistant (MDR) and extensively drug-resistant (XDR) pathogens are being reported globally [1]. Both MDR and XDR gram-negative rods (GNRs) have been defined by the Centers for Disease Control and Prevention (CDC) and the European Center for Disease Prevention and Control as acquired non-susceptibility to at least one drug in three or more antimicrobial categories and to all but two or less antimicro bial categories, respectively [2]. Additional clinical definitions have been suggested, such as difficult-to-treat (DTT) infections [3], defined as antimicrobial resistance among GNRs including *Escherichia coli*, *Klebsiella* spp., *Enterobacter* spp., *Pseudomonas aeruginosa*, and *Acinetobacter baumannii* that limits treatment with all safe, highly efficacious, first-line antibacterials, such as β-lactams, including carbapenems and β-lactam/β- lactamase inhibitor combinations, fluoroquinolones, and extended-spectrum cephalosporins such as ceftazidime, cefepime, and ceftriaxone [3]. To be considered DTT, an isolate must be tested against at least one agent from each of these three categories [3]. By definition, all DTT pathogens should be MDR, and many should also be XDR [3]. Regardless of the definition, MDR/XDR/DTT pathogens are associated with substantial morbidity, mortality, and health care costs [4]. Non-susceptibility to first-line agents delays the time to effective therapy, which is considered the only modifiable risk factor for mortality in patients with MDR/XDR/DTT gram-negative pathogens [4]. This, in turn, compels the use of alternative, more toxic antimicrobials [5]. Among the new antibiotics, meropenem/vaborbactam, imipenem/relebactam/cilastatin, and cefiderocol represent valid therapeutic options against DTT pathogens [6].

Colistin is an old-revived antimicrobial agent with a significant efficacy against gram-negative pathogens [5]. Its unique mechanism of action helps to retain its activity against MDR and XDR bacteria regardless of the mechanism of resistance [5]. Colistin has been used increasingly as a last-resort treatment for MDR gram-negative pathogens. However, concerns about nephrotoxicity with intravenous polymixins using conventional doses continue to be reported in addition to ongoing variable pharmacokinetic-pharmacodynamic data, thus limiting their use as an initial drug of choice [5]. These agents are usually reserved for XDR strains and avoided if alternative agents with adequate gram-negative activity are available [5]. In addition, while there is some clinical evidence indicating the superiority of monotherapy of colistin over its combination, for management of carbapenem-resistant P. aeruginosa (CRPA), as an example [7], similar clinical and mortality outcomes were observed with colistin monotherapy versus colistin combined with meropenem in patients with carbapenem-resistant Acinetobacter baumannii (CRAB) pathogens as another example [8].

In the new era of antibiotics, tigecycline is an established second-generation tetracycline-class antibacterial agent developed for the treatment of polymicrobial MDR pathogens, including gram-positive and gram-negative organisms [9]. While the Food and Drug Administration issued in 2010 an alert that tigecycline significantly increased the risk of mortality in the management of patients with severe infections [10], and with the rise of drug resistance among *Enterobacteriaceae* through the production of several beta-lactamases, tigecycline becomes a potential option given its ability to evade common mechanisms of acquired tetracycline resistance [8]. Additionally, given the early warnings about high mortality rates, especially when given as monotherapy, combination therapy with tigecycline seems essential [11].

Overall, colistin and tigecycline are therapeutic options for the MDR and XDR pathogens, and the efficacy and safety of both agents remain controversial. There are no summative quantitative summaries in the literature that specifically evaluate colistin versus tigecycline use in various infections, and even though there are newer drugs, colistin use is still recommended as the first line for some MDR/XDR pathogens (e.g., pneumonia) caused by Acinetobacter baumanii. Hence, in this study, we aimed to perform a comparative meta-analysis of the main efficacy and safety outcomes of colistin versus tigecycline, as both monotherapies or combinations, in treating infections caused by MDR and XDR pathogens. 

## 2. Results

### 2.1. Search Strategy Results

**Figure 1:**
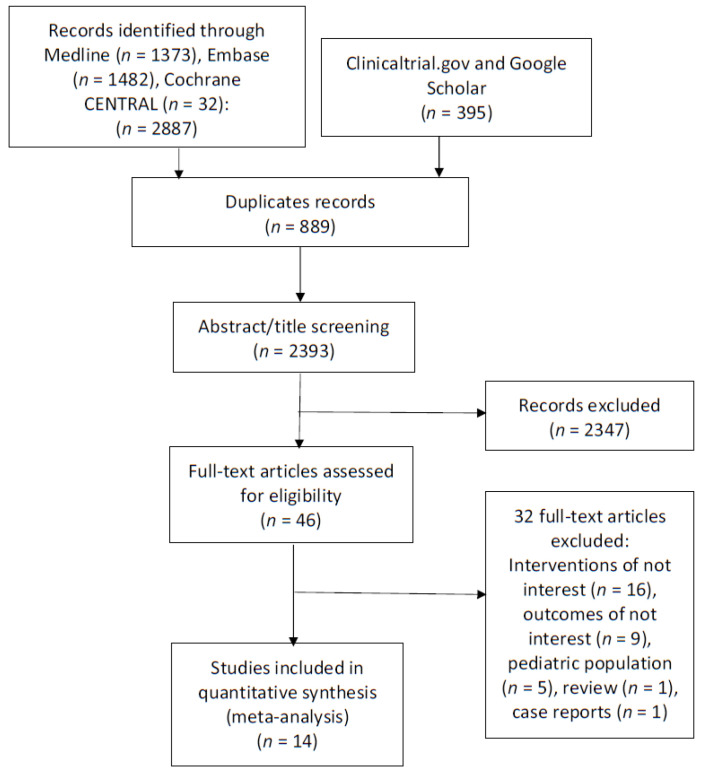
PRISMA flow diagram.

Our literature search yielded 2392 unique records. After title and abstract screening, 46 studies were retrieved and screened for eligibility. The flow of the study selection process is shown in the Preferred Reporting Items for Systematic Reviews and Meta-Analyses (PRISMA) flow diagram in Figure 1.

### 2.2. Characteristics of Included Studies

Fourteen studies [7,12,13,14,15,16,17,18,19,20,21,22,23,24] involving 1163 patients were included in the current study. A total of 789 patients were treated with tigecycline and 938 patients were treated with colistin. The mean duration of treatment ranged from 8.3 to 42.5 days. Countries included were Greece (*n* = 3), Taiwan (*n* = 3), Korea (*n* = 3), and Spain (*n* = 2), and one study was conducted in each of the United States (US), Brazil, and Thailand. All articles were published in English between 1982 and 2021. A summary of the design and baseline characteristics of enrolled patients is presented in Table 1. All included studies were associated with a low risk of bias according to the Newcastle–Ottawa Scale (NOS) checklist that could be found in Table S1.

### 2.3. Outcomes

#### 2.3.1. Tigecycline Monotherapy versus Colistin Monotherapy

**Figure 2:**
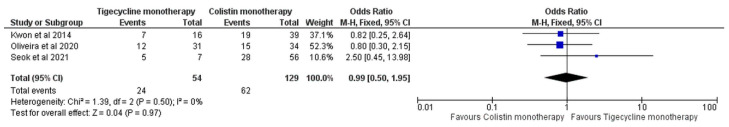
Clinical response of tigecycline monotherapy versus colistin monotherapy [12,17,23].

Three studies [12,17,23] reported on clinical response, and the effect estimate showed no significant difference between the compared groups (odds ratio (OR) = 0.99, 95% confidence interval (CI) [0.50, 1.95], *p* = 0.97). No significant heterogeneity was found (I^2^ = 0%, *p* = 0.50), Figure 2.

**Figure 3:**
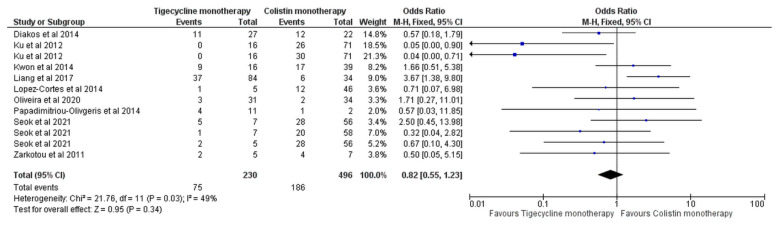
Overall mortality of tigecycline monotherapy versus colistin monotherapy [7,12,14,15,16,17,19,23,24].

Nine studies [7,12,14,15,16,17,19,23,24] reported the overall mortality of tigecycline monotherapy versus colistin monotherapy. The effect estimate did not favour either drug (OR = 0.82, 95% CI [0.55, 1.23], *p* = 0.34), Figure 3. No significant heterogeneity was observed (I^2^ = 49%, *p* = 0.03). As seen by the funnel plot analysis in Figure S1.1, no publication bias was observed. 

**Figure 4:**
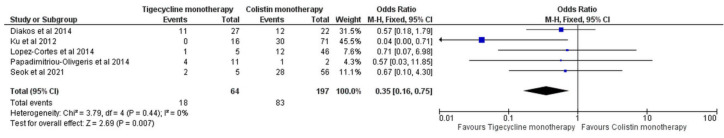
Thirty-day mortality of tigecycline monotherapy versus colistin monotherapy [7,14,15,17,24].

The 30-day mortality was reported in five studies [7,14,15,17,24], where tigecycline monotherapy was significantly associated with less mortality than colistin monotherapy (OR = 0.35, 95% CI [0.16, 0.75], *p* = 0.007), Figure 4. No significant heterogeneity was observed (I^2^ = 0%, *p* = 0.44).

**Figure 5:**
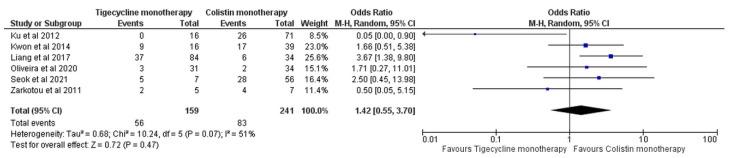
In-hospital mortality of tigecycline monotherapy versus colistin monotherapy [12,15,16,17,19,23].

In contrast, for the in-hospital mortality, reported in six studies [12,15,16,17,19,23], no statistically significant advantage was reported with either monotherapy (OR = 1.42, 95% CI [0.55, 3.70], *p* = 0.47), Figure 5, with a significant heterogeneity observed (I ^2^ = 51%, *p* = 0.07). However, when reversing the heterogeneity via the sensitivity analysis, by excluding the study by Ku et al. [15], a statistically reduced in-hospital mortality rate was observed in favour of the colistin monotherapy (OR = 2.27, 95% CI [1.24, 4.16], *p* = 0.008), with no heterogeneity (I^2^ = 0%, *p* = 0.57), Figure S1.2.

Recurrence of infection was only reported in one study [12], where no difference between the study monotherapies was reported (OR = 1.39, 95% CI [0.38, 5.12], *p* = 0.62), Figure S1.3. 

The 7-day mortality was also reported in one study only [17], where no difference existed (OR = 0.32, 95% CI [0.04, 2.82], *p* = 0.3), Figure S1.4. The 14-day mortality was not reported in any of the relevant studies. 

Renal impairment estimates did not differ between the colistin and tigecycline monotherapies, reported in four studies [12,17,22,23] (OR = 0.41, 95% CI [0.11, 1.52], *p* = 0.18), Figure S1.5, including significant heterogeneity (I^2^ = 69%, *p* = 0.02). When the heterogeneity was reduced, however, by excluding the study by Seok et al. [17], renal impairment became statistically significantly reduced with tigecycline (OR = 0.24, 95% CI [0.12, 0.47], *p* < 0.0001), Figure S1.6, (I^2^ = 0%, *p* = 0.79). For hepatic enzymes abnormalities, and with no significant heterogeneity (I^2^ = 0%, *p* = 0.33), there was no statistical difference between the colistin and tigecycline monotherapies, based on two studies [12,17] (OR = 3.21, 95% CI [0.74, 13.95], *p* = 0.12), Figure S1.7.

#### 2.3.2. Monotherapy versus Combination Therapy

**Figure 6:**
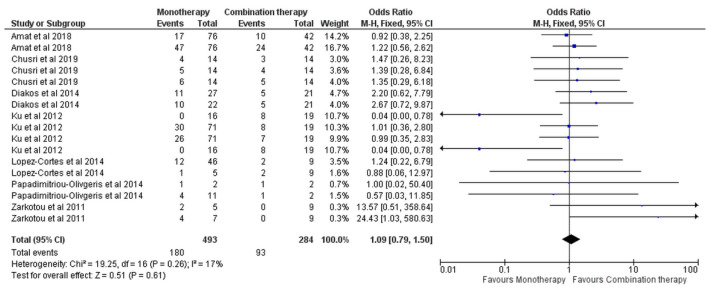
Clinical response of monotherapy versus combination monotherapy [7,13,14,15,16,18,24].

No estimates were reported for clinical response. For the overall mortality, based on seven studies [7,13,14,15,16,18,24], the effect estimates were comparable between the monotherapies and the colistin plus tigecycline combination (OR = 1.09, 95% CI [0.79, 1.50], *p* = 0.61), Figure 6, with no significant heterogeneity observed (I^2^ = 17%, *p* = 0.26). The funnel plot analysis did not show publication bias, Figure S1.8.

**Figure 7:**
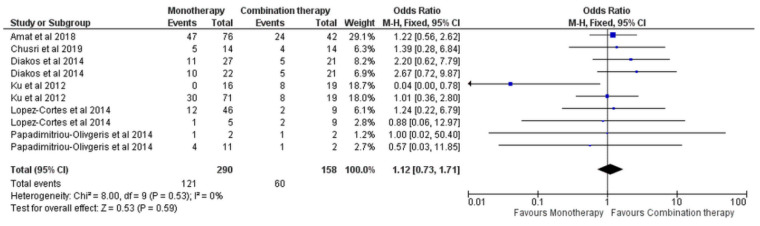
Thirty-day mortality of monotherapy versus combination monotherapy [7,13,14,15,18,24].

The 30-day mortality also did not differ between the monotherapies and the combination, as reported by six studies [7,13,14,15,18,24] (OR = 1.12, 95% CI [0.73, 1.71], *p* = 0.59), Figure 7. No significant heterogeneity was observed (I^2^ = 0%, *p* = 0.53). Possible publication bias was observed, Figure S1.9, and when this was improved by excluding the subgroup of Ku et al. [15], with OR = 0.04, 95% CI [0.00, 0.78], the outcome did not change (OR = 1.37, 95% CI [0.88, 2.14], *p* = 0.17).

**Figure 8:**
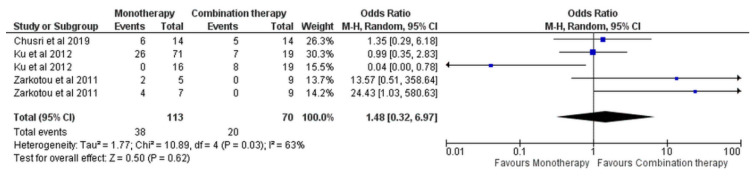
In-hospital mortality of monotherapy versus combination monotherapy [13,15,16].

For the in-hospital mortality, a similar trend of no difference between the monotherapies and the combination was observed, based on three studies [13,15,16] (OR = 1.48, 95% CI [0.32, 6.97], *p* = 0.62), Figure 8, with significant heterogeneity (I^2^ = 63%, *p* = 0.03). Via sensitivity analysis and the exclusion of the subgroup of Ku et al. [15], with OR = 0.04, 95% CI [0.00, 0.78], the outcome did not change (OR = 1.80, 95% CI [0.85, 3.84], *p* = 0.13), Figure S1.10.

No studies reported the recurrence or the 7-day mortality rates, but two studies [13,18] reported the 14-day mortality, Figure S1.11, where no significant difference was observed (OR = 1.02, 95% CI [0.46, 2.25], *p* = 0.96), with no significant heterogeneity (I^2^ = 0%, *p* = 0.64). 

Renal impairment was reported in only one study [13], where the outcome significantly favoured the monotherapy over the combination (OR = 0.11, 95% CI [0.02, 0.61], *p* = 0.01), Figure S1.12. No relevant studies reported hepatic enzyme abnormalities estimates.

#### 2.3.3. Tigecycline Combination versus Colistin Combination

**Figure 9:**
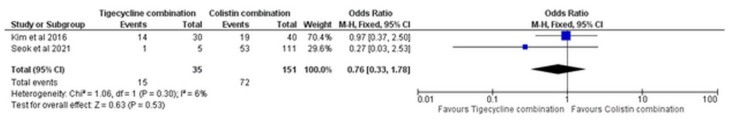
Clinical response of tigecycline combination versus colistin combination [17,20].

Only two studies reported the clinical response of colistin combination versus tigecycline combination [17,20], where the results did not favour a combination over another (OR = 0.76, 95% CI [0.33, 1.78], *p* = 0.53), Figure 9. No significant heterogeneity was observed (I^2^ = 6%, *p* = 0.30).

**Figure 10:**
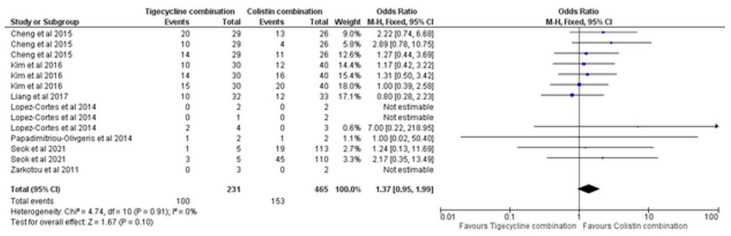
Overall mortality of tigecycline combination versus colistin combination [14,16,17,19,20,21,24].

The overall mortality was based on seven studies [14,16,17,19,20,21,24], and no statistical difference was calculated between the study combinations (OR = 1.37, 95% CI [0.95, 1.99], *p* = 0.10), Figure 10, with no significant heterogeneity (I^2^ = 0%, *p* = 0.91). No publication bias was observed by the funnel plot analysis, Figure S1.13.

**Figure 11:**
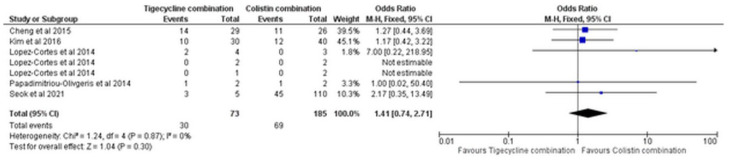
Thirty-day mortality of tigecycline combination versus colistin combination [14,17,20,21,24].

The 30-day mortality did not also differ between the study combinations, based on five studies [14,17,20,21,24] (OR = 1.41, 95% CI [0.74, 2.71], *p* = 0.30), Figure 11, with no significant heterogeneity (I^2^ = 0%, *p* = 0.87). 

**Figure 12:**
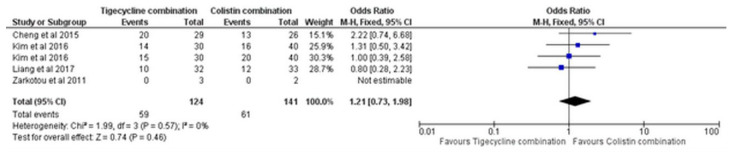
In-hospital mortality of tigecycline combination versus colistin combination [16,19,20,21].

Similarly, based on four studies [16,19,20,21], the in-hospital mortality did not statistically significantly differ between the colistin combination versus tigecycline combination (OR = 1.21, 95% CI [0.73, 1.98], *p* = 0.46), Figure 12, with no significant heterogeneity (I^2^ = 0%, *p* = 0.57).

Recurrence of infection, 7-day mortality, and 14-day mortality were each reported in one study [17,20,21], where no combination was favourable (OR = 0.64, 95% CI [0.11, 3.77], *p* = 0.62, Figure S1.14, (OR = 1.24, 95% CI [0.13, 11.69], *p* = 0.85), Figure S1.15, and (OR = 2.89, 95% CI [0.78, 10.75], *p* = 0.11), Figure S1.16, respectively.

Based on three studies [17,20,21], renal impairment was significantly reduced with the tigecycline combination over the colistin combination (OR = 0.32, 95% CI [0.11, 0.92], *p* = 0.03), Figure S1.17, with no observed significant heterogeneity (I^2^ = 8%, *p* = 0.34). The hepatic enzymes abnormalities were reported in one study [17], and no statistical difference was observed between both combinations (OR = 1.52, 95% CI [0.16, 14.44], *p* = 0.72), Figure S1.18.

#### 2.3.4. Subgroup Analysis

##### Tigecycline Monotherapy versus Colistin Monotherapy

Similar to the clinical response at base case, subgroup analysis of clinical cure only, excluding clinical improvement, did not show any statistical difference between colistin and tigecycline monotherapies, based on three studies [12,17,23] OR = 0.81, 95% CI [0.38, 1.72], *p* = 0.58), Figure S2.1, with no significant heterogeneity (I^2^ = 0%, *p* = 0.98).

Four studies analysed 28-day and 30-day mortalities only [7,14,17,24], excluding 3-month mortality [15], and similar to the base case, a statistical difference between the monotherapies was not observed (OR = 0.61, 95% CI [0.26, 1.44], *p* = 0.26), Figure S2.2. No significant heterogeneity was observed (I^2^ = 0%, *p* = 1.00).

For all-cause in-hospital mortality, excluding intensive care unit (ICU) [19] and infection-caused mortalities [16], there was also no statistical difference between the study monotherapies, based on four studies [12,16,17,23] (OR = 1.10, 95% CI [0.28, 4.30], *p* = 0.89), Figure S2.3, with an observed significant heterogeneity (I^2^ = 57%, *p* = 0.07), which when reversed by excluding the study by Ku et al. [15], did not change the outcome (OR = 1.86, 95% CI [0.79, 4.39], *p* = 0.15), with no heterogeneity (I^2^ = 0%, *p* = 0.93).

Looking at nephrotoxicity alone in three studies [17,22,23], in isolation from other renal impairments [12], none of the monotherapies demonstrated any significant advantage (OR = 0.69, 95% CI [0.09, 5.33], *p* = 0.72), with significant heterogeneity (I^2^ = 77%, *p* = 0.01), Figure S2.4. Eliminating heterogeneity by excluding the study by Seok et al. [17], however, and just like with the renal impairment at base case, a statistically significant reduction in nephrotoxicity with tigecycline monotherapy was calculated as compared with colistin (OR = 0.27, 95% CI [0.12, 0.64], *p* = 0.003), with insignificant heterogeneity (I^2^ = 0%, *p* = 0.58).

##### Monotherapy versus Combination Therapy

For the 30-day mortality, comparing the colistin plus tigecycline combination against the colistin monotherapy alone did not show a significant advantage to either, based on six studies [7,13,14,15,18,24] (OR = 1.31, 95% CI [0.78, 2.21], *p* = 0.30), with no significant heterogeneity (I^2^ = 0%, *p* = 0.84), Figure S2.5. A similar trend with the 30-day mortality was also observed when comparing the colistin plus tigecycline combination against the tigecycline monotherapy only, based on six studies [7,13,14,15,18,24] (OR = 0.82, 95% CI [0.39, 1.71], *p* = 0.30), with no significant heterogeneity (I^2^ = 41%, *p* = 0.15), Figure S2.6.

Only analysing 28-day and 30-day mortalities in five studies [7,13,14,18,24], excluding 3-month mortality [14], did not change the base case result of no significant difference between monotherapy and the combination (OR = 1.40, 95% CI [0.86, 2.30], *p* = 0.18), Figure S2.7. No significant heterogeneity was observed (I^2^ = 0%, *p* = 0.84).

Identical to the subgroup analyses of 30-day mortality, the outcomes with the base case in-hospital mortality did not also change with the subgroup analyses. Only looking at the colistin monotherapy calculated an OR = 3.46, 95% CI [0.15, 78.21], *p* = 0.43, Figure S2.8, with significant heterogeneity (I^2^ = 73%, *p* = 0.06), based on two studies [13,16]. Eliminating heterogeneity by excluding either study does not change the study outcome. Only looking at the tigecycline monotherapy for the in-hospital mortality calculated an OR = 0.89, 95% CI [0.05, 15.23], *p* = 0.94, Figure S2.9 with significant heterogeneity (I^2^ = 73%, *p* = 0.02), based on three studies [13,15,16]. Reducing heterogeneity by excluding a study group in the study by Ku et al. [15] did not change the outcome (OR = 2.68, 95% CI [0.34, 21.34], *p* = 0.35), with no significant heterogeneity (I^2^ = 37%, *p* = 0.21).

A subgroup analysis of two studies of all-cause in-hospital mortality [13,15], excluding infection-caused mortality [16], did not change the base case outcome (OR = 0.62, 95% CI [0.14, 2.82], *p* = 0.54), Figure S2.10, with significant heterogeneity (I^2^ = 60%, *p* = 0.08). Eliminating heterogeneity by excluding a study group in the study by Ku et al. [15] did not change the outcome (OR = 1.09, 95% CI [0.46, 2.60], *p* = 0.84), with no significant heterogeneity (I^2^ = 0%, *p* = 0.74).

##### Tigecycline Combination versus Colistin Combination

The only subgroup analysis possible for the colistin combination versus tigecycline combination was for the in-hospital mortality, excluding ICU mortality. In two studies [19,20], the in-hospital mortality, excluding ICU mortality, was not different between both combinations (OR = 1.41, 95% CI [0.69, 2.86], *p* = 0.35), Figure S2.11. No significant heterogeneity was observed (I^2^ = 14%, *p* = 0.28).

### 2.4. Quality of Evidence

Overall, based on the grading of recommendation, assessment, development, and evaluation (GRADE) approach of assessment, the study outcomes were associated with moderate to high-quality evidence. None of the outcomes was associated with a low or very low level of quality. Details of the GRADE quality of evidence can be found in Table S2.

## 3. Discussion

Currently, there are still no evidence-based guidelines recommending which antimicrobial therapy options the clinicians should consider to treat infections caused by MDR and XDR pathogens, whereby clinicians should think through all clinically relevant patient-specific factors such as renal and hepatic functions, site of infection, other comorbidities, and local pattern of drug resistance for decision making [25]. The rapidly emerging antimicrobial resistance poses a critical challenge in managing MDR/XDR pathogens. New antibiotics have recently been launched targeting the most problematic gram-negative pathogens, such as CRPA and carbapenem-resistant *Enterobacteriaceae* (CRE). However, these should not be considered the solution to the ongoing crisis of MDR/XDR pathogens. The revived antibiotic, colistin, is still considered because it has a fundamental role in the treatment of CRE as monotherapy or in combination with other antibiotics, in the treatment of CRPA as well as in the treatment of CRAB. Additionally, when CRAB is prevalent, tigecycline or colistin are the only currently available options [26].

There are meta-analyses that evaluated whether colistin or tigecycline in combination with other antibiotics are associated with better outcomes compared with colistin or tigecycline monotherapy in patients with MDR and XDR pathogens [27,28]. However, studies that analysed colistin versus tigecycline as monotherapy or combination against MDR and XDR pathogens are lacking. In our meta-analysis, there was no significant difference between colistin and tigecycline, whether given as monotherapies, or in combination with each other or with other antimicrobials, in achieving better response rates or reducing reinfection rates in patients with MDR/XDR gram-negative pathogens. The overall mortality rates did not also differ among all comparator arms. The 30-day mortality rate, however, was lower with tigecycline monotherapy as compared with colistin monotherapy. The in-hospital mortality ratewas lower in colistin monotherapy compared with tigecycline monotherapy. Here, in our study, most included patients treated with tigecycline were managed in the ICU and had sepsis and ventilator-associated pneumonia, which may have contributed to the relatively high in-hospital mortality rates compared with those treated with colistin [7,13,14,17,18,19,21]. 

The renal impairment was lower with tigecycline compared to colistin, whether given as monotherapy or in combination with other therapies. For the monotherapy use, the tigecycline advantage also included the nephrotoxicity event occurrence in isolation. No significant differences were reported for the hepatic impairment.

Multiple risk factors for colistin-associated renal toxicity are described in the literature. These include advanced age, weight extremes, chronic comorbidities, pre-existing renal impairment, low albumin levels, and concomitant nephrotoxic agents [29]. In our analysis, in at least 10 studies, the median age of participants receiving colistin therapy was 60 years and above. In addition, patients in these studies had several predisposing factors, such as diabetes mellitus, chronic kidney disease, cardiovascular diseases, hypovolemia, taking nephrotoxic drugs, and admission to the critical care units. Two recent studies showed higher odds of nephrotoxicity with colistin therapy compared with b-lactam and tigecycline-based regimens in treating gram-negative pathogens [30,31].

Antibiotic options to treat CRE infections are limited. Previous treatment options included salvage antibiotics with preserved in vitro activity such as aminoglycosides, polymyxins, and tigecycline, which are limited by variable rates of in vitro activity, high rates of toxicity, suboptimal pharmacokinetics, and poor effectiveness when used as monotherapy [32]. Accordingly, combination therapies have been used to overcome the limitations of any agent individually [32]. Polymyxins are recommended in combination with an additional agent that is susceptible to invasive infections caused by MDR/XDR *A. baumannii* infections [8]. Nevertheless, based on the literature, the combination therapy for MDR and XDR pathogens remains controversial. In a multicentre observational study, giving colistin in combination with other antimicrobials was associated with a survival benefit over monotherapy alone [8]. In another study, however, the combination of tigecycline with carbapenem showed a comparable outcome versus tigecycline monotherapy, while the combination of colistin with carbapenem showed a less favorable outcome compared to colistin monotherapy [33]. This was supported by a recent meta-analysis by Cheng et al., which revealed that colistin combination therapy was not associated with a lower mortality rate compared to monotherapy [34].

Regarding the colistin plus tigecycline combination, our study failed to show any benefit of using the combination over monotherapies. In fact, our results demonstrated that the combination therapy of colistin plus tigecycline seems to be associated with increased renal impairment compared to the monotherapy use, i.e., colistin.

In a recent meta-analysis by Kengkla et al., where patients with MDR and XDR pathogens, who received a combination of colistin and other antibiotics, were significantly associated with lower all-cause mortality than those who received a combination of sulbactam and other antibiotics [25]. However, based on our study, for how the colistin versus tigecycline combination with other therapies compare, none of the combinations demonstrated efficacy advantages, including with response rate, mortality, and reoccurrence. To note, however, in a meta-analysis of tigecycline use for MDR *A. baumannii* pneumonia infections based on three studies that evaluated in-hospital mor tality, tigecycline combination was associated with a higher rate of mortality (OR 1.57; 95% CI, 1.04–2.35; *p* = 0.03) compared with the colistin combination [34]. Although tigecycline has been used for *Acinetobacter* infections, PK-PD limitations must be considered when other treatment options are available. Because of its high volume of distribution, its usefulness for bloodstream infections is not currently recommended [35]. Newer drugs such as ceftazidime/avibactam have shown good clinical outcomes and better survival outcomes when compared with colistin-based combinations for CRE infections [5,36]. Additionally, meropenem/vaborbactam, which has recently been demonstrated to be a potential therapeutic strategy against ceftazidime/avibactam-resistant *Klebsiella pneumoniae* when combined with ATM [37], showed better clinical cure rates compared to colistin- or tigecycline-based combinations in treating patients with CRE infections [38].

With interest in Pseudomonas aeruginosa as a common MDR pathogen, and given the lack of specific data in the included studies, it is not possible to stratify analysis for Pseudomonas aeruginosa as an etiological agent. We found that only Liang et al., reported the outcomes of the presence of Pseudomonas aeruginosa [19]. According to this, the number of death in patients receiving tigecycline monotherapy was higher compared with those receiving colistin monotherapy (*n* = 37 versus 6). For the monotherapy against combination, the mortality rate was higher in patients receiving tigecycline monotherapy (*n* = 37) compared with those receiving colistin or tigecycline combined with other antimicrobials (*n* = 12 and 10, respectively) or colistin monotherapy (*n* = 6). For the combination therapy, more death cases were reported with the colistin combination compared to the tigecycline combination (*n* = 12 versus 10). It is a strength that this is the first head-to-head meta-analysis between colistin and tigecycline in the literature. This included subgroup and sensitivity analyses to account for heterogeneity and specific characteristics among study participants. However, this meta-analysis has several limitations. First, only a limited number of studies met the inclusion criteria, and not all included studies reported all outcomes of interest. In addition, the included studies are limited to observational studies of small sample sizes. Here, potential bias and confounders are inherent, which may introduce uncertainty. To add, although the heterogeneity among studies in analyses was generally insignificant, and the sensitivity analysis was performed when otherwise, variations between studies concerning, for example, concurrent antimicrobials, time to initiate therapy, disease severity, and the study site may affect the robustness of interpretations made. Furthermore, we only included studies published in English, which may have led to missing some relevant literature studies. Additionally, while the literature search in this study is comprehensive, it is always possible that additional search terms and/or searching additional grey literature sources may identify additional studies. Finally, the authors of the included studies were not contacted when reported data were lacking. As per the GRADE assessment, however, no serious imprecision existed in the included studies.

Overall, within the limitations of this study, it seems that there are no differences between colistin and tigecycline monotherapies against MDR and XDR pathogens in terms of clinical cure and reinfection rate. The tigecycline monotherapy, however, was associated with lower rates of 30-day mortality and renal toxicity, while the colistin was associated with lower in-hospital mortality. The colistin-tigecycline combination had no advantage over monotherapies, and apart from lower renal toxicity with the tigecycline combination with other therapies, this did not have any benefit for any outcome against the colistin combination.

## 4. Materials and Methods

This meta-analysis was performed in accordance with the Cochrane handbook of systematic reviews of interventions, and followed the PRISMA guidelines [39], Table S3. The study protocol was published online at the PROSPERO International Prospective Register of Systematic Reviews, registration number: CRD42021286063.

### 4.1. Literature Search Strategy

We searched PubMed, Cochrane CENTRAL, and EMBASE for comparative studies (retrospective or prospective) comparing tigecycline monotherapy or combination versus colistin monotherapy or combination, from inception until May 15, 2021. We used the possible combinations of the keywords, including tigecycline, tygacil, glycylcycline, TGC, colistin, bactaeremia, bloodstream infection, sepsis, septicaemia, multidrug-resistant, MDR, carbapenem-resistant, extensively drug resistant, XDR, *Acinetobacter baumannii*, *Klebsiella pneumoniae*, *Enterobacteriaceae*, and CRE, including variant spellings and endings. ClinicalTrials.gov and Google Scholar were also searched, with similar search terms. Additionally, the reference lists of identified articles were checked manually. Detailed search strategies can be found Table S4.

### 4.2. Study Selection and Data Extraction

We included comparative studies (retrospective or prospective) that compared tigecycline monotherapy or combination versus colistin monotherapy or combination for treating patients with MDR or XDR gram-negative pathogens. We excluded animal models, reviews, case reports, case series, non-English articles, and duplicate references. 

We conducted eligibility screening in two steps, each by two independent reviewers, via title and abstract screening first, and then the full-text screening for eligibility to meta-analysis. Two independent authors extracted requisite data into a data extraction form. The extracted data included items about baseline characteristics of enrolled patients, general characteristics of study design, and included outcomes. Disagreements between reviewers were discussed with a third reviewer and resolved upon consensus.

### 4.3. Outcomes of Meta-Analysis

The outcomes of interest were efficacy and safety outcomes. The primary outcomes were the response rate and the mortality, including overall, 30-day, and in-hospital mortalities. Secondary outcomes were recurrence, 7-day and 14-day mortalities, renal impairment, and hepatic enzymes abnormalities.

Response rate is defined as clinical cure (resolution of symptoms and signs of infection by the end of therapy) and/or clinical improvement (partial resolution of symptoms and signs of infection). Overall mortality was defined as the compilation of the 7-day, 14-day, 30-day, and in-hospital mortalities. 30-day mortality was defined to include all-cause 28-day, 30-day, and 3-month mortalities. In-hospital mortality was defined as all-cause mortality until discharge, including ICU mortality and infection-caused mortality. Recurrence is the recurrence of infection after response. The 7-day and 14-day mortalities are all-cause mortalities. The renal impairment was identified as reported in included studies, and it included nephrotoxicity. The hepatic enzyme abnormality was also identified as reported in included studies, and it included hepatotoxicity.

Colistin and tigecycline outcomes were categorised under three comparative groups: colistin monotherapy versus tigecycline monotherapy; colistin monotherapy and/or tigecycline monotherapy versus the combination of colistin plus tigecycline; and the colistin versus tigecycline combination with identical other therapies. 

### 4.4. Risk of Bias Assessment

NOS was used to assess the quality of observational studies. Each included study was assessed based on reporting of three essential domains: (i) selection of the study subjects, (ii) comparability of groups on demographic characteristics and critical potential confounders, and (iii) ascertainment of the prespecified outcome (exposure/treatment) [40]. Two reviewers assessed the risk of bias, with an additional third reviewer to resolve any disagreements.

### 4.5. Statistical Analysis

Data for analysis in this study were dichotomous data and were pooled as OR, with 95% CI. We used RevMan™ 5.3 software provided by the Cochrane Collaboration (Cochrane Collaboration, Copenhagen, Denmark) for data synthesis [41]. Meta-analyses were conducted to combine data across studies on the magnitude and direction of treatment effects for each study outcome separately. Significant statistical heterogeneity was indicated by Q statistic *p*-value less than 0.1 or by $I^{2}$ more than 50%. In case of significant heterogeneity, a random effect model was employed. Otherwise, the fixed effect model was used. Furthermore, based on the methods reported in the Cochrane Handbook for Systematic Reviews of Interventions, when a study included multiple groups for an intervention, such as different monotherapies versus the combination, where all groups fit the inclusion criteria in the pooled analysis, the multiple groups were included in the analysis as independent comparisons to avoid double-counting and the unit-of-analysis error [42]. For a pooled analysis that includes zero events in studies, the Mantel–Haenszel (MH) method was used, via RevMan™, and without zero cell correction if the zero cell did not exist in all studies included in an analysis. The MH method excludes studies where both arms are zero events [41,43].

A trial sequential analysis (TSA) was not performed. The TSA is to assess type I errors due to systematic errors (bias) or random errors of randomised controlled trials. Given the observational (non-randomised) nature of included studies, the results of TSA will be biased (not accurate) and will limit the conclusion of the study [44]. We conducted sensitivity analyses by excluding one study at a time to assess any sources of heterogeneity. Publication bias was assessed using the funnel plot for outcomes from more than ten studies.

Subgroup analyses were performed to analyse (i) clinical cure only, without clinical improvement, (ii) the 28/30-day mortality alone (without 3-month mortality), (iii) the all-cause in-hospital mortality, without the ICU and infection-caused mortalities, (iv) the colistin monotherapy alone versus its combination with tigecycline, (v) the tigecycline monotherapy alone versus its combination with colistin, (vi) nephrotoxicity, and (vii) hepatotoxicity, where two or more studies were available for analysis.

### 4.6. Quality of Evidence

Two reviewers independently assessed the strength of recommendations and evidence provided by the pooled results using the GRADE Handbook. This looks at risk of bias, inconsistency, indirectness, imprecision, and publication bias with the overall levels of quality classified as “high”, “moderate”, “low”, or “very low” [45].

## 5. Conclusions

There was no significant difference between colistin and tigecycline, whether given as monotherapies or as combinations with each other or with other antimicrobials, in achieving better response rates, reducing reinfection rates or reducing overall mortality rates in patients with MDR/XDR gram-negative pathogens. Compared with colistin, however, the 30-day mortality rate was lower with the tigecycline monotherapy, and the renal toxicity was lower with the tigecycline combination. Future well-designed controlled trials and/or analyses of real-life data registered are still necessary to confirm the comparative efficacy and safety of both antimicrobials.

## Figures and Tables

**Table 1:** Baseline characteristics and summary of included studies.

Author, Year	Study Design, Period	Country	Age (Mean ± Standard Deviation or Range), (Tigecycline/Colistin)	Site of Infection	Causative Pathogens	Sample Size (Tigecycline/Colistin)	Concomitant Antibiotics in Tigecycline Group	Concomitant Antibiotics in Colistin Group	Mean Duration of Therapy in Days (Tigecycline vs. Colistin)	Tigecycline Regimen	Colistin Regimen	Clinical Outcomes Reported	Baseline Charlson Comorbidity Index (Tigecycline vs. Colistin)	Major Comorbidities
Oliveira et al., 2020	Retrospective cohort study, 2007–2015	Brazil	46.8 ± 18.9/40.6 ± 19.1	Osteomyelitis in non-critically ill patients	CRABc	31/34	NA	NA	42 vs. 42.5	Continuous dose: 50 mg intravenous every 12 h	Continuous dose: 2.5 mg/kg intravenous every 12 h	Remission, recurrence of infection, in-hospital mortality, liver enzymes abnormalities, renal impairment	Mean (range): 1 (0; 7) vs. 0 (0; 5)	Hypertension, diabetes, neoplasia, rheumatoid arthritis, HIV, immunodeficiency
Chusri et al., 2019	Retrospective cohort study, 2012–2017	Thailand	45/46	IAI in non-and critically ill patients	CRAB	14/14	Colistin	NA	21 vs. 22	Loading dose: 100 mg Continuous dose:50 mg every 12 h	Loading dose: 5 mg/kg or 300mg Continuous dose: 2.5 mg/kg/dose	Microbiological eradication; mortality at 14 days, 30 days, in hospital mortality; renal complications	NR	NR
Amat et al., 2018	Retrospective cohort study,2010–2012	Spain	56 ± 13/58 ± 17	Mixed in critically ill patients	CRAB	42/76	Colistin	NA	14 vs. 14	Loading dose 100 mg Continuous dose: 50 mg every 12 h	Continuous dose: 9 million IU every 24 h	Mortality at 14 days and 30 days	Mean (SD): 2.7 ± 2.4 vs. 2.6 ± 1.8	Diabetes, COPD, immunosuppressed, liver cirrhosis, CKD
Liang et al., 2018	Retrospective cohort study, 2010–2015	Taiwan	73.7 (62.8–82.0) (all patients)	Pneumonia in critically ill patients	CRAB- pneumonia	159/67	Meropenem, imipenem, sulbactam, colistin	Meropenem, imipenem, sulbactam	14 vs. 14	As per Sanford guide	As per Sanford guide	ICU and hospital mortality, treatment failure, recurrence	Total mean (range): 3 (2–4) (all patients)	Acute respiratory failure, septic shock, gastrointestinal bleeding, post operation
Kim et al., 2016	Retrospective cohort study, 2009–2010	Korea	72 (64–76)/67 (57–75)	Mixed in critically ill patients	MDR/XDRAB pneumonia	30/40	Carbapenem, sulbactam, sulbactam+ minocycline+ rifampicin, rifampicin doxycycline	Carbapenem alone or with combination, sulbactam, rifampicin, sulbactam +rifampicin doxycycline	11 vs. 12	Loading dose: 100 mg Continuous dose: 50 mg every 12 h	Loading dose: 5 mg/kg Continuous dose:150 mg every 12 h	Clinical success, microbiology success, recurrence of infection, hospital mortality at 30 day, ICU mortality, in-hospital mortality, nephrotoxicity	NR	Hypertension, chronic pulmonary, kidney, liver diseases, diabetes, cancer, immunodeficiency
Cheng et al., 2015	Prospective cohort, 2010–2013	Taiwan	62 vs. 62	Bacteremia in critically ill patients	XDDRAb	29/26	Colistin	Carbapenem	10 vs. 9 (median)	Loading dose:100 mg Continuous dose: 50 mg every 12 h	2.5–5 mg/kg/day divided every 8 or 12 h	All cause mortality at 14 days, all cause in-hospital mortality, 30 days and on discharge, nephrotoxicity	median (IQR): 4 (3–5) vs. 3 (1–6)	CLD, CKD, lung, diseases, cancer, diabetes, stroke, liver cirrhosis, transplantation, CHF
Chuang et al., 2014	Retrospective cohort study, 2009–2010	Taiwan	63.8 ± 17.9/63.7 ± 19.5	Mixed in critically ill patient	MDRAB pneumonia	175/119	Aminoglycoside, sarbapenem, sulbactam	Aminoglycoside, sarbapenem, sulbactam	13.1 vs. 14.6	Loading dose: 100 mg Continuous dose:50 mg every 12 h.	2.5–5 mg/kg/day divided doses every 8 or 12 h	In-hospital mortality, nephrotoxicity	NR	CVD, diabetes, CKD, liver cirrhosis, CLD, cancer, immunosuppressed
Daikos et al., 2014	Retrospective cohort study, 2009–2010	Greece	62.7 ± 17.5 (all patients)	BSI, non- andcritically ill patients	CP-Kp	NR	Aminoglycoside, carbapenem	Aminoglycoside carbapenem	NR	100–200 mg every 12h	9 million IU every 8–12 h	All-cause mortality at 28 day	NR	Neutropenia, sepsis
Kwon et al., 2014	Retrospective cohort study, 2009–2010	Korea	60.1 ± 12.3/59.0 ± 19.2	Mixed in non- and critically ill patients	XDRAB	16/39	NA	NA	13 vs. 15	50–100 mg every 24 h	75–300 mg every 24 h	Clinical success, in-hospital mortality, side effects as nephrotoxicity	NR	Hypertension, kidney and liver diseases, diabetes, tuberculosis
Lopez-Cortes et al., 2014	Prospective cohort study, 2010	Spain	60 (52–75) (all patients, median (IQR))	Mixed in non- and critically ill patients	MDRAB	5/46 (monotherapy) 22/23 (combination)	Colistin, carbapenem, aminoglycoside, rifampicin	Tigecycline, carbapenem, sulbactam, aminoglycoside	NR	NR	NR	Mortality at 14 and 30 day	NR	Diabetes, CLD, cancer, dialysis, immunodeficiency
Papadimitriou-Olivgeris et al., 2014	Retrospective cohort study, NA	Greece	55.2 ± 19.3 (all patients)	BSI in critically ill patients	KPC-Kp	16\93	NR	NR	NR	NR	NR	Mortality at 30 day	NR	Diabetes, COPD, cancer, CVD and CKD
Ku et al., 2012	Retrospective study, 2009	USA	56.9 ± 19.1/60.2 ± 18.3	Mixed in non- and critically ill patients	CRE	16/71 monotherapy 19 (colistin-tigecycline combination)	Colistin	Tigecycline	8.6 vs.. 8.3	NR	NR	In hospital mortality and mortality in 3 months	Mean (SD): 3.4 ± 3.1 vs. 3.5 ± 3	Diabetes, CVD. CLD, cancer, chronic liver disease, neurologic disease,
Zarkotou et al., 2011	Prospective cohort study, 2008–2010	Greece	63.8 ± 19.9 (all patients)	BSI in non- and critically ill patients	KPC-Kp	5/7 (monotherapy) 17/2 (combination)	Colistin, gentamicin, carbapenem, amikacin	Gentamicin	NR	NR	NR	Microbiological response, failure, and Indeterminate, mortality in 14 days and all-cause in-hospital mortality, BSI-mortality	NR	NR
Seok et al., 2021	Prospective cohort study, 2015–2016	Korea	67.0 ± 14.9 (all patients)	Mixed in critically ill patients	CRAB	12/171	NA	Carbapenem, minocycline, rifampin, sulbactam,	NA	NA	NA	Mortality at 7 and 28-day, clinical success, microbiocidal response at 14 and 28-day, nephrotoxicity and hepatoxicity	NA	Diabetes, CVD, neuromuscular diseases, CKD, liver disease, COPD, asthma, transplantation

MDRAB, multi-drug resistant *Acinetobater baumannii*; XDRAB, extensively drug resistant *Acinetobater baumannii* complex; CRABc, carbapenem-resistant *Acinetobater baumannii* complex; KPC-kp, *Klebsiella pneumoniae* carbapenemases-producing *K. pneumoniae*; CRE, carbapenem-resistant *Enterobacteriaceae*; BSI, blood stream infection; IAI, intraabdominal infection; NA: not applicable; NR, not reported; SD, standard deviation; VAP: Ventilator-associated pneumonia; UTI: Urinary tract infection; HIV: human immunodeficiency virus; COPD: chronic obstructive pulmonary disease; CKD: chronic kidney disease; IU: international unit; ICU: intensive care unit; CLD: chronic lung disease; CVD: cardiovascular disease.

## Data Availability

The data presented in this study are available in the article and the Supplementary Materials.

## Supplementary Materials

The following supporting information can be downloaded at: https://www.mdpi.com/article/10.3390/antibiotics11111630/s1, Table S1: Methodological quality of the included cohort observational studies; Table S2: Quality of evidence for the included outcomes; Table S3: PRISMA 2020 checklist; Table S4: Search strategies; Figure S1: Additional base case analysis results; Figure S2. Subgroup analysis results.
