# Supplementary material for: Efficacy and Safety of Colistin versus Tigecycline for Multi-Drug-Resistant and Extensively Drug-Resistant Gram-Negative Pathogens—A Meta-Analysis
Source: Antibiotics (Basel). 2022 Nov 15;11(11):1630. doi: 10.3390/antibiotics11111630 (PMC9686723; doi:10.3390/antibiotics11111630)

Table S1: Methodological quality of the included cohort observational studies based on the New Castle Ottawa scale for assessing the quality of epidemiological studies.

Study	Selection				Comparability	Outcome			Total Score
	Representativeness of the exposed cohort	Selection of the non-exposed cohort	Ascertainment of exposure[1]	Outcome was not present at start of study[2]	Control for 2 important factors[3.4]	Assessment of outcome	Follow-up long enough	Adequacy of follow-up of cohort[7]	
Oliveira et al 2020	*	*	*	-	*	*	*	*	7
Chusri et al 2019	*	*	*	-	*	*	*	*	7
Amat et al 2018	*	*	*	-	*	*	*	*	7
Liang et al 2017	*	*	*	-	*	*	*	*	7
Kim et al 2016	*	*	*	-	*	*	*	*	7
Cheng et al 2015	*	*	*	*	*	*	*	*	8
Chaung et al 2014	*	*	*	-	*	*	*	*	7
Diakos et al 2014	*	*	*	-	*	*	*	*	7
Kwon et al 2014	*	*	*	-	*	*	*	*	7
Lopez-Cortes et al 2014	*	*	*	*	**	*	*	*	9
Papadimitriou-Olivgeris et al 2014	*	*	*	-	*	*	*	*	7
Zarkotou et al 2011	*	*	*	*	*	*	*	*	8
Ku et al 2012	*	*	*	-	*	*	*	*	7

Seok et al 2021	*	*	-	*	*	*	*	*	7

[1] If the exposure data was obtained from prescription database or medical record, a point was assigned.

[2] If the study design is prospective study, a point was assigned.

[3] If adjusted for age, a point was assigned.

[4] If adjusted for any other additional factors, a point was assigned.

[5] If the completeness of follow-up was 80% or more, a point was assigned.

Table S2: Quality of evidence for the included outcomes.

Outcome	Number of studies	GRADE quality assessment					
		Risk of bias	Inconsistency	Indirectness	Imprecision	Publication bias	Overall quality of evidence
**Tigecycline monotherapy versus colistin monotherapy**							
Clinical response	3	Low	No series inconsistency	No series indirectness	No series imprecision	Undetected	⊕⊕⊕⊕ High
Overall mortality	9	Low	No series inconsistency	No series indirectness	No series imprecision	Undetected	⊕⊕⊕⊕ High
30-day mortality	5	Low	No series inconsistency	No series indirectness	No series imprecision	Undetected	⊕⊕⊕⊖ Moderate
In-hospital mortality	6	Low	Series inconsistency (due to significant heterogeneity)	No series indirectness	No series imprecision	Undetected	⊕⊕⊕⊖ Moderate
7-day mortality	1	Low	No series inconsistency	No series indirectness	No series imprecision	Undetected	⊕⊕⊕⊕ High
Recurrence of infection	1	Low	No series inconsistency	No series indirectness	No series imprecision	Undetected	⊕⊕⊕⊕ High

Renal impairment	4	Low	Series inconsistency (due to significant heterogeneity)	No series indirectness	No series imprecision	Undetected	⊕⊕⊕⊖ Moderate
Hepatic enzymes abnormalities	2	Low	No series inconsistency	No series indirectness	No series imprecision	Undetected	⊕⊕⊕⊕ High
**Monotherapy versus combination therapy**							
Overall mortality	7	Low	No series inconsistency	No series indirectness	No series imprecision	Undetected	⊕⊕⊕⊕ High
30-day mortality	6	Low	No series inconsistency	No series indirectness	No series imprecision	Detected	⊕⊕⊕⊖ Moderate
In-hospital mortality	3	Low	Series inconsistency (due to significant heterogeneity)	No series indirectness	No series imprecision	Undetected	⊕⊕⊕⊖ Moderate
14-day mortality	2	Low	No series inconsistency	No series indirectness	No series imprecision	Undetected	⊕⊕⊕⊕ High
Renal impairment	1	Low	No series inconsistency	No series indirectness	No series imprecision	Undetected	⊕⊕⊕⊕ High
**Tigecycline combination and colistin combination**							
Clinical response	2	Low	No series inconsistency	No series indirectness	No series imprecision	Undetected	⊕⊕⊕⊕ High

Overall mortality	7	Low	No series inconsistency	No series indirectness	No series imprecision	Undetected	⊕⊕⊕⊕ High
30-day mortality	5	Low	No series inconsistency	No series indirectness	No series imprecision	Undetected	⊕⊕⊕⊕ High
In-hospital mortality	4	Low	No series inconsistency	No series indirectness	No series imprecision	Undetected	⊕⊕⊕⊕ High
7-day mortality	1	Low	No series inconsistency	No series indirectness	No series imprecision	Undetected	⊕⊕⊕⊕ High
14-day mortality	1	Low	No series inconsistency	No series indirectness	No series imprecision	Undetected	⊕⊕⊕⊕ High
Recurrence of infection	1	Low	No series inconsistency	No series indirectness	No series imprecision	Undetected	⊕⊕⊕⊕ High
Renal impairment	3	Low	No series inconsistency	No series indirectness	No series imprecision	Undetected	⊕⊕⊕⊕ High
Hepatic enzymes abnormalities	1	Low	No series inconsistency	No series indirectness	No series imprecision	Undetected	⊕⊕⊕⊕ High

Table S3: PRISMA Checklist.

Section and Topic	Item #	Checklist item
**TITLE**		
Title	1	Identify the report as a systematic review.
**ABSTRACT**		
Abstract	2	See the PRISMA 2020 for Abstracts checklist.
**INTRODUCTION**		
Rationale	3	Describe the rationale for the review in the context of existing knowledge.
Objectives	4	Provide an explicit statement of the objective(s) or question(s) the review addresses.
**METHODS**		
Eligibility criteria	5	Specify the inclusion and exclusion criteria for the review and how studies were grouped for the syntheses.
Information sources	6	Specify all databases, registers, websites, organisations, reference lists and other sources searched or consulted to identify studies. Specify thedate when each source was last searched or consulted.
Search strategy	7	Present the full search strategies for all databases, registers and websites, including any filters and limits used.
Selection process	8	Specify the methods used to decide whether a study met the inclusion criteria of the review, including how many reviewers screened each recordand each report retrieved, whether they worked independently, and if applicable, details of automation tools used in the process.
Data collection process	9	Specify the methods used to collect data from reports, including how many reviewers collected data from each report, whether they worked independently, any processes for obtaining or confirming data from study investigators, and if applicable, details of automation tools used in theprocess.
Data items	10a	List and define all outcomes for which data were sought. Specify whether all results that were compatible with each outcome domain in eachstudy were sought (e.g. for all measures, time points, analyses), and if not, the methods used to decide which results to collect.
	10b	List and define all other variables for which data were sought (e.g. participant and intervention characteristics, funding sources). Describe any assumptions made about any missing or unclear information.
Study risk of bias assessment	11	Specify the methods used to assess risk of bias in the included studies, including details of the tool(s) used, how many reviewers assessed each study and whether they worked independently, and if applicable, details of automation tools used in the process.
Effect measures	12	Specify for each outcome the effect measure(s) (e.g. risk ratio, mean difference) used in the synthesis or presentation of results.
Synthesis methods	13a	Describe the processes used to decide which studies were eligible for each synthesis (e.g. tabulating the study intervention characteristics andcomparing against the planned groups for each synthesis (item #5)).
	13b	Describe any methods required to prepare the data for presentation or synthesis, such as handling of missing summary statistics, or data conversions.
	13c	Describe any methods used to tabulate or visually display results of individual studies and syntheses.

	13d	Describe any methods used to synthesize results and provide a rationale for the choice(s). If meta-analysis was performed, describe themodel(s), method(s) to identify the presence and extent of statistical heterogeneity, and software package(s) used.
	13e	Describe any methods used to explore possible causes of heterogeneity among study results (e.g. subgroup analysis, meta-regression).
	13f	Describe any sensitivity analyses conducted to assess robustness of the synthesized results.
Reporting bias assessment	14	Describe any methods used to assess risk of bias due to missing results in a synthesis (arising from reporting biases).
Certainty assessment	15	Describe any methods used to assess certainty (or confidence) in the body of evidence for an outcome.

Table S4: Search strategies

**Database: Medline <1950 to 2021 May 15>**
Search Strategy:
1 tigecycline or tygacil or glycylcycline or TGC, or 'GAR-936'or tigecycline monotherapy, tigecycline combination.mp. (4583)
2 Colistin or colistin monotherapy or colistin combination.mp. (2881)
3 1 and 2 (903)
4 Bactaeremia or bacteremia or bloodstream infection or sepsis or septicaemia/ (57014)
5 Multidrug-resistant or MDR or carbapenem-resistant or extensively drug resistant or XDR or carbapenem combination therapy/ (55730)
6 Acinetobacter baumannii or Klebsiella pneumoniae or Pseudomonas aeruginosa or carbapenemase producing or Enterobacteriaceaemp or carbapenem-resistant Enterobacteriaceae, or CRE (17414)
7 3 and (4 or 5 or 6) (30195)
8 observational*.tw. (229)
9 Comparative study*.tw. (1404)
10 clinical trial*.tw. (198)
11 randomized controlled trial/ (153)
12 controlled clinical trial*.tw. (21)
13 or/7-12 (1870)
14 7 and 13 (1455)
15 limit 14 to (Human and English language) (1373)
**Database: EBM Reviews - Cochrane Central Register of Controlled Trials <April 2020>**
Search Strategy:
1 tigecycline or tygacil or glycylcycline or TGC, or 'GAR-936'or tigecycline monotherapy, tigecycline combination (title abstract keyword) (238)
2 Colistin or colistin monotherapy or colistin combination (title abstract keyword) (462)
3 1 and 2 (35)
4 Bactaeremia or bacteremia or bloodstream infection or sepsis or septicaemia or Multidrug-resistant or MDR or carbapenem-resistant or extensively drug resistant or XDR or carbapenem combination therapy or Acinetobacter baumannii or Klebsiella pneumoniae or Pseudomonas aeruginosa or carbapenemase producing or Enterobacteriaceaemp or carbapenem-resistant Enterobacteriaceae, or CRE (18189)
5 3 and 4 (32)
**Database: Embase <1974 to May 15, 2021>**
Search Strategy:

1 tigecycline or tygacil or glycylcycline or TGC, or ‘GAR-936’or tigecycline monotherapy, tigecycline combination.mp. (5342)
2 Colistin or colistin monotherapy or colistin combination.mp. (3468)
3 1 and 2 (843)
4 Bactaeremia or bacteremia or bloodstream infection or sepsis or septicaemia/ (62865)
5 Multidrug-resistant or MDR or carbapenem-resistant or extensively drug resistant or XDR or carbapenem combination therapy/ (69521)
6 Acinetobacter baumannii or Klebsiella pneumoniae or Pseudomonas aeruginosa or carbapenemase producing or Enterobacteriaceaemp or carbapenem-resistant Enterobacteriaceae, or CRE (21535)
7 3 and 4 or 5 or 6 (41532)
8 observational*.tw. (351)
9 Comparative study*.tw. (1605)
10 clinical trial*.tw. (262)
11 randomized controlled trial/ (167)
12 controlled clinical trial*.tw. (46)
13 or/7-12 (2154)
14 7 and 13 (1563)
15 limit 14 to (Human and English language) (1482)
**Grey literature sources: clinicaltrial.gov, and Google Scholar < May 15, 2021>**
Search Strategy:
All possible combinations among search terms from each of the following domains were manually created and searched. Only a single term from each domain was used in any combination.
- Drug 1 domain: tigecycline or tygacil or glycylcycline or TGC, or ‘GAR-936’, tigecycline monotherapy, tigecycline combination.
- Drug 2 domain: colistin, colistin monotherapy, colistin combination
- Condition domain: Bacteremia or bacteremia or bloodstream infection or sepsis or septicaemia or Multidrug-resistant or MDR or carbapenem-resistant or extensively drug resistant or XDR or carbapenem combination therapy or Acinetobacter baumannii or Klebsiella pneumoniae or Pseudomonas aeruginosa or carbapenemase producing or Enterobacteriaceaemp or carbapenem-resistant Enterobacteriaceae, or CRE.
Grey literature sources: clinicaltrial.gov, and Google Scholar < May 15, 2021>
Search Strategy:
All possible combinations among search terms from each of the following domains were manually created and searched. Only a single term from each domain was used in any combination.

- Drug 1 domain: tigecycline or tygacil or glycylcycline or TGC, or ‘GAR-936’, tigecycline monotherapy, tigecycline combination.
- Drug 2 domain: colistin, colistin monotherapy, colistin combination
- Condition domain: Bacteremia or bacteremia or bloodstream infection or sepsis or septicaemia or Multidrug-resistant or MDR or carbapenem-resistant or extensively drug resistant or XDR or carbapenem combination therapy or Acinetobacter baumannii or Klebsiella pneumoniae or Pseudomonas aeruginosa or carbapenemase producing or Enterobacteriaceaemp or carbapenem-resistant Enterobacteriaceae, or CRE.

Supplementary Figure Materials S1. Additional base case analysis results

Figure S1.1. Funnel plot for overall mortality: colistin versus tigecycline monotherapies

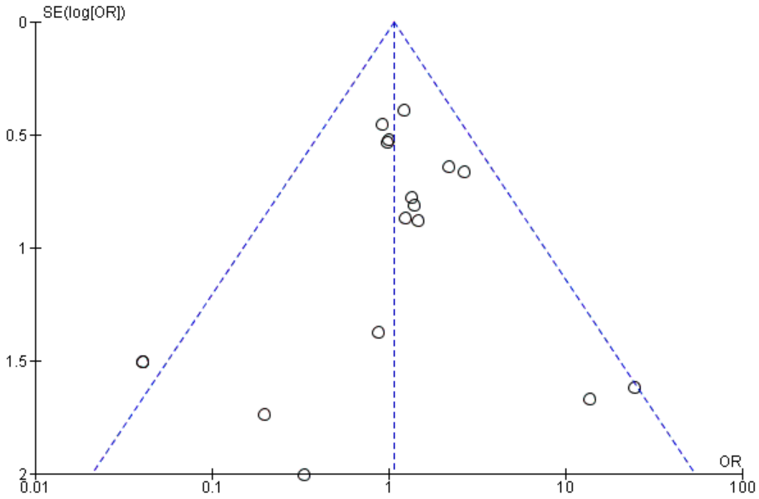

Figure S1.2. Sensitivity analysis for in-hospital mortality: colistin versus tigecycline monotherapies

	Tigecycline monotherapy		Colistin monotherapy			Odds Ratio
Study or Subgroup	Events	Total	Events	Total	Weight	M-H, Fixed, 95% CI
Ku et al 2012	0	16	26	71	0.0%	0.05 [0.00, 0.90]
Kwon et al 2014	9	16	17	39	29.6%	1.66 [0.51, 5.38]
Liang et al 2017	37	84	6	34	32.7%	3.67 [1.38, 9.80]
Oliveira et al 2020	3	31	2	34	11.8%	1.71 [0.27, 11.01]
Seok et al 2021	5	7	28	56	12.2%	2.50 [0.45, 13.98]
Zarkotou et al 2011	2	5	4	7	13.7%	0.50 [0.05, 5.15]
**Total (95% CI)**		**143**		**170**	**100.0%**	**2.27 [1.24, 4.16]**
Total events	56		57			

Heterogeneity: Chi² = 2.91, df = 4 (P = 0.57); I² = 0%
Test for overall effect: Z = 2.65 (P = 0.008)

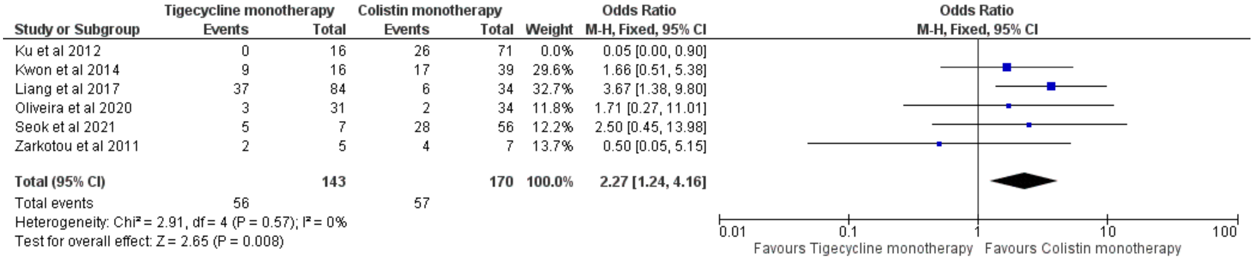

Figure S1.3. Recurrence: colistin versus tigecycline monotherapies

	Tigecycline monotherapy		Colistin monotherapy			Odds Ratio
Study or Subgroup	Events	Total	Events	Total	Weight	M-H, Fixed, 95% CI
Oliveira et al 2020	6	31	5	34	100.0%	1.39 [0.38, 5.12]
**Total (95% CI)**		**31**		**34**	**100.0%**	**1.39 [0.38, 5.12]**
Total events	6		5			

Heterogeneity: Not applicable
Test for overall effect: Z = 0.50 (P = 0.62)

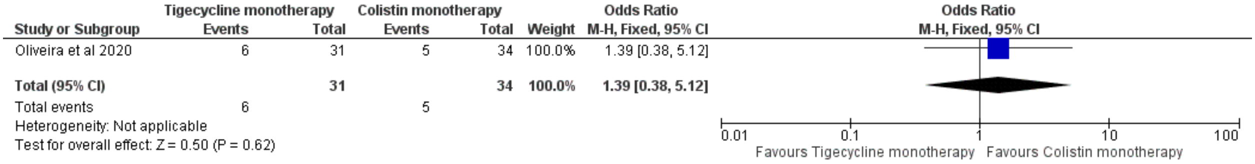

Figure S1.4. 7-day mortality: colistin versus tigecycline monotherapies

	Tigecycline monotherapy		Colistin monotherapy			Odds Ratio
Study or Subgroup	Events	Total	Events	Total	Weight	M-H, Fixed, 95% CI
Seok et al 2021	1	7	20	58	100.0%	0.32 [0.04, 2.82]
**Total (95% CI)**		**7**		**58**	**100.0%**	**0.32 [0.04, 2.82]**
Total events	1		20			

Heterogeneity: Not applicable
Test for overall effect: Z = 1.03 (P = 0.30)

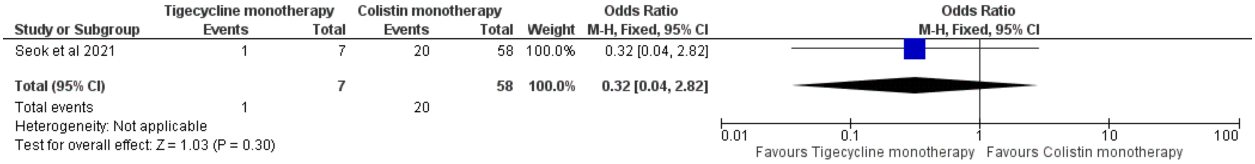

Figure S1.5. Renal impairment: colistin versus tigecycline monotherapies

	Tigecycline monotherapy		Colistin monotherapy			Odds Ratio
Study or Subgroup	Events	Total	Events	Total	Weight	M-H, Random, 95% CI
Chaung et al 2014	6	175	12	119	31.6%	0.32 [0.12, 0.87]
Kwon et al 2014	2	16	17	39	24.4%	0.18 [0.04, 0.93]
Oliveira et al 2020	7	31	20	34	30.7%	0.20 [0.07, 0.60]
Seok et al 2021	7	7	26	58	13.2%	18.40 [1.00, 337.13]
**Total (95% CI)**		**229**		**250**	**100.0%**	**0.41 [0.11, 1.52]**
Total events	22		75			

Heterogeneity: Tau² = 1.12; Chi² = 9.66, df = 3 (P = 0.02); I² = 69%
Test for overall effect: Z = 1.33 (P = 0.18)

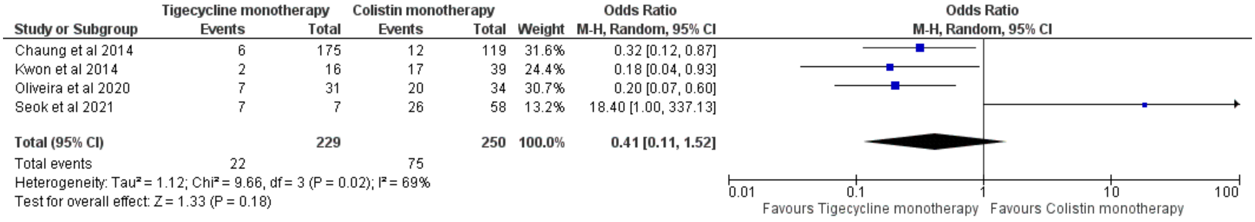

Figure S1.6. Sensitivity analysis for renal impairment: colistin versus tigecycline monotherapies

	Tigecycline monotherapy		Colistin monotherapy			Odds Ratio
Study or Subgroup	Events	Total	Events	Total	Weight	M-H, Fixed, 95% CI
Chaung et al 2014	6	175	12	119	37.1%	0.32 [0.12, 0.87]
Kwon et al 2014	2	16	17	39	23.3%	0.18 [0.04, 0.93]
Oliveira et al 2020	7	31	20	34	39.7%	0.20 [0.07, 0.60]
Seok et al 2021	7	7	26	58	0.0%	18.40 [1.00, 337.13]
**Total (95% CI)**		**222**		**192**	**100.0%**	**0.24 [0.12, 0.47]**
Total events	15		49			

Heterogeneity: Chi² = 0.47, df = 2 (P = 0.79); I² = 0%
Test for overall effect: Z = 4.16 (P < 0.0001)

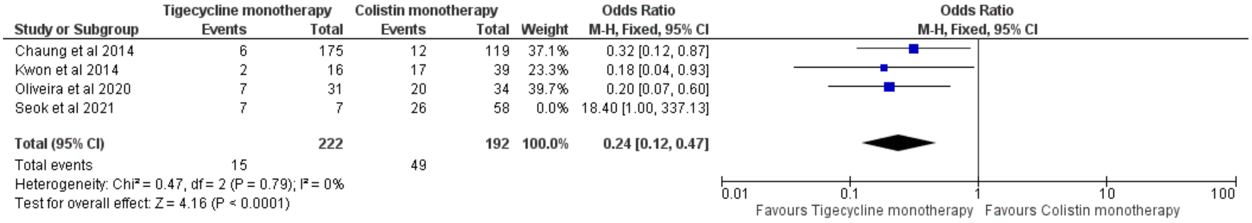

Figure S1.7. Liver enzymes abnormalities: colistin versus tigecycline monotherapies

	Tigecycline monotherapy		Colistin monotherapy			Odds Ratio
Study or Subgroup	Events	Total	Events	Total	Weight	M-H, Fixed, 95% CI
Oliveira et al 2020	1	31	1	34	51.7%	1.10 [0.07, 18.37]
Seok et al 2021	3	7	7	58	48.3%	5.46 [1.01, 29.69]
**Total (95% CI)**		**38**		**92**	**100.0%**	**3.21 [0.74, 13.95]**
Total events	4		8			

Heterogeneity: Chi² = 0.94, df = 1 (P = 0.33); I² = 0%
Test for overall effect: Z = 1.55 (P = 0.12)

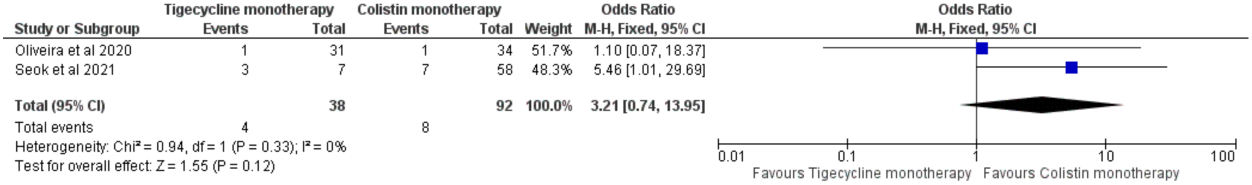

Figure S1.8. Funnel plot for overall mortality: monotherapy versus combination

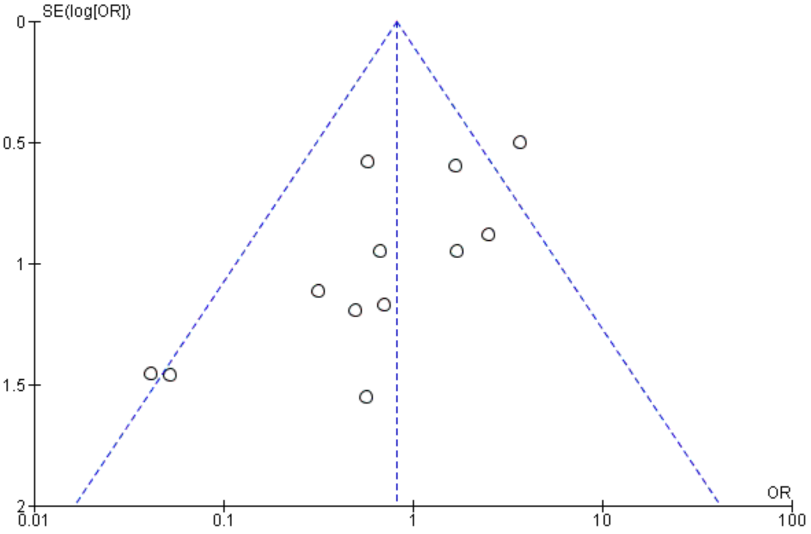

Figure S1.9. Funnel plot for 30-day mortality: monotherapy versus combination

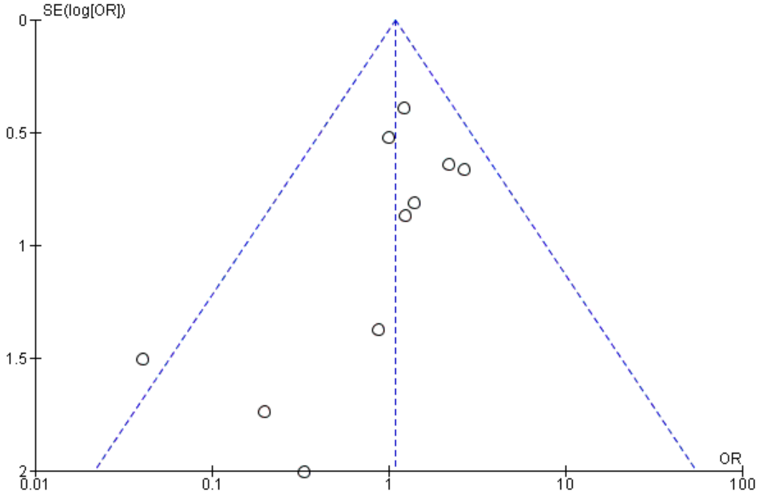

Figure S1.10. Sensitivity analysis for in-hospital mortality: monotherapy versus combination

Study or Subgroup	Monotherapy Events	Monotherapy Total	Combination therapy Events	Combination therapy Total	Weight	Odds Ratio M-H, Fixed, 95% CI
Chusri et al 2019	6	14	5	14	27.8%	1.35 [0.29, 6.18]
Ku et al 2012	26	71	7	19	68.2%	0.99 [0.35, 2.83]
Ku et al 2012	0	16	8	19	0.0%	0.04 [0.00, 0.78]
Zarkotou et al 2011	2	5	0	9	2.1%	13.57 [0.51, 358.64]
Zarkotou et al 2011	4	7	0	9	1.9%	24.43 [1.03, 580.63]
**Total (95% CI)**		**97**		**51**	**100.0%**	**1.80 [0.85, 3.84]**
Total events	38		12			

Heterogeneity: Chi² = 5.45, df = 3 (P = 0.14); I² = 45%
Test for overall effect: Z = 1.53 (P = 0.13)

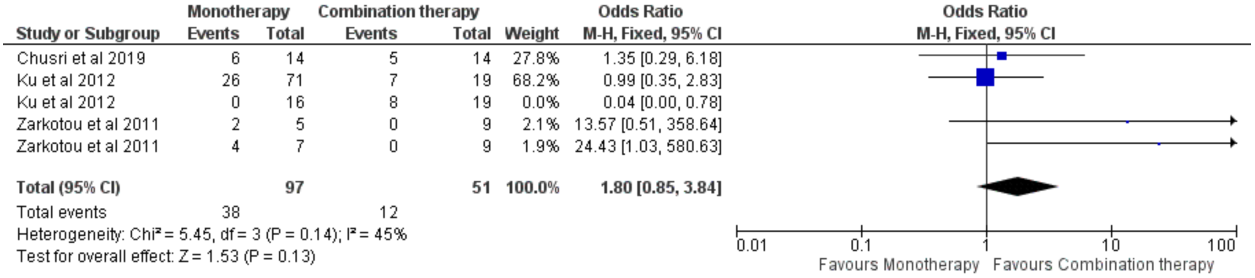

Figure S1.11. 14-day mortality: monotherapy versus combination

Study or Subgroup	Monotherapy Events	Monotherapy Total	Combination therapy Events	Combination therapy Total	Weight	Odds Ratio M-H, Fixed, 95% CI
Amat et al 2018	17	76	10	42	82.4%	0.92 [0.38, 2.25]
Chusri et al 2019	4	14	3	14	17.6%	1.47 [0.26, 8.23]
**Total (95% CI)**		**90**		**56**	**100.0%**	**1.02 [0.46, 2.25]**
Total events	21		13			

Heterogeneity: Chi² = 0.22, df = 1 (P = 0.64); I² = 0%
Test for overall effect: Z = 0.04 (P = 0.96)

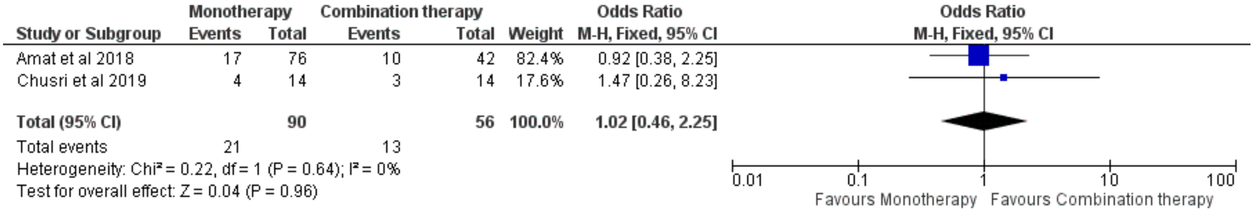

Figure S1.12. Renal impairment: monotherapy versus combination

Study or Subgroup	Monotherapy Events	Monotherapy Total	Combination therapy Events	Combination therapy Total	Weight	Odds Ratio M-H, Fixed, 95% CI
Chusri et al 2019	3	14	10	14	100.0%	0.11 [0.02, 0.61]
**Total (95% CI)**		**14**		**14**	**100.0%**	**0.11 [0.02, 0.61]**
Total events	3		10			

Heterogeneity: Not applicable
Test for overall effect: Z = 2.52 (P = 0.01)

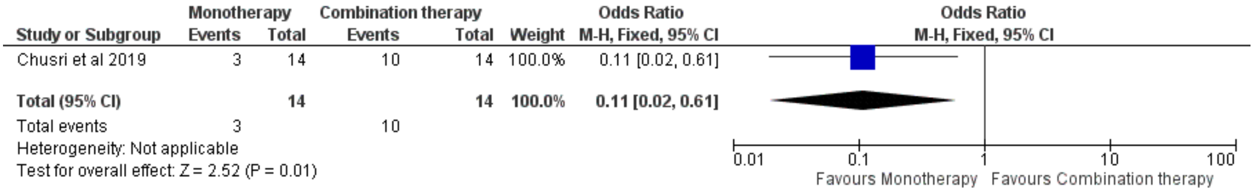

Figure S1.13. Funnel plot for overall mortality: colistin versus tigecycline combinations

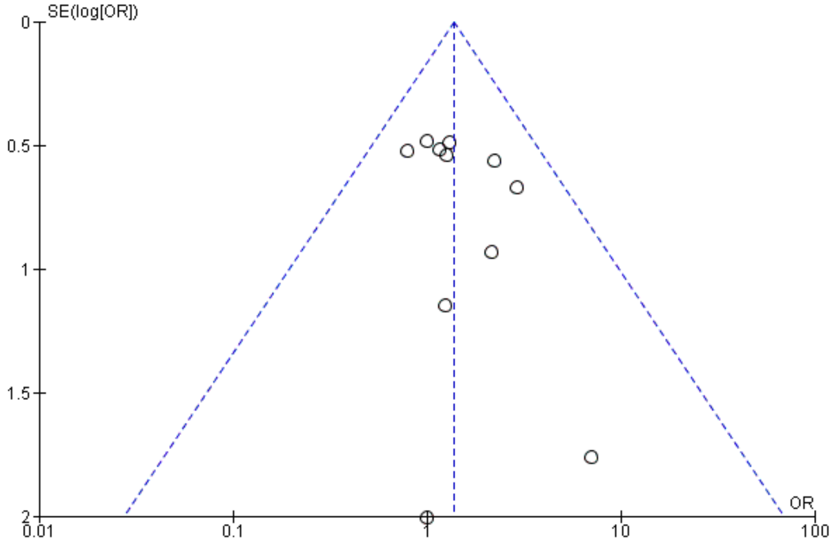

Figure S1.14. Recurrence: colistin versus tigecycline combinations

	Tigecycline combination		Colistin combination			Odds Ratio
Study or Subgroup	Events	Total	Events	Total	Weight	M-H, Fixed, 95% CI
Kim et al 2016	2	30	4	40	100.0%	0.64 [0.11, 3.77]
**Total (95% CI)**		**30**		**40**	**100.0%**	**0.64 [0.11, 3.77]**
Total events	2		4			
Heterogeneity: Not applicable						
Test for overall effect: Z = 0.49 (P = 0.62)						

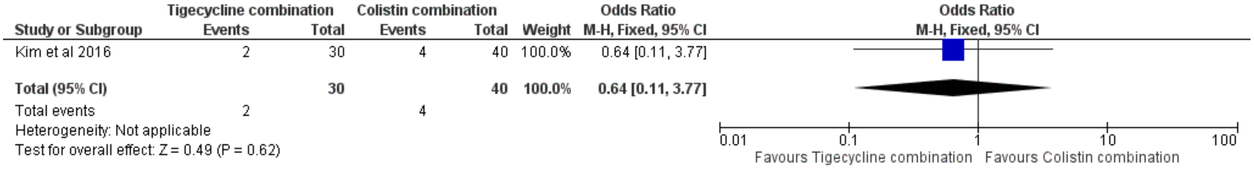

Figure S1.15. 7-day mortality: colistin versus tigecycline combinations

	Tigecycline combination		Colistin combination			Odds Ratio
Study or Subgroup	Events	Total	Events	Total	Weight	M-H, Fixed, 95% CI
Seok et al 2021	1	5	19	113	100.0%	1.24 [0.13, 11.69]
**Total (95% CI)**		**5**		**113**	**100.0%**	**1.24 [0.13, 11.69]**
Total events	1		19			
Heterogeneity: Not applicable						
Test for overall effect: Z = 0.19 (P = 0.85)						

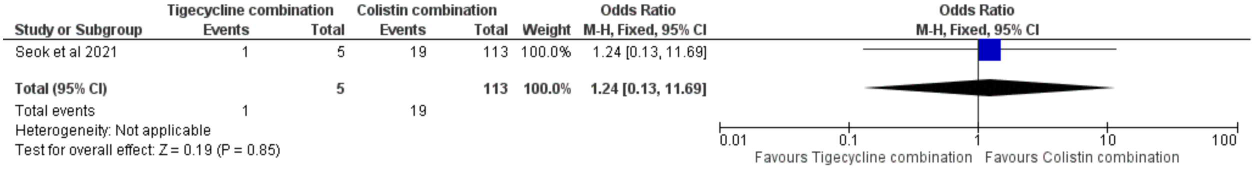

Figure S1.16. 14-day mortality: colistin versus tigecycline combinations

	Tigecycline combination		Colistin combination			Odds Ratio
Study or Subgroup	Events	Total	Events	Total	Weight	M-H, Fixed, 95% CI
Cheng et al 2015	10	29	4	26	100.0%	2.89 [0.78, 10.75]
**Total (95% CI)**		**29**		**26**	**100.0%**	**2.89 [0.78, 10.75]**
Total events	10		4			
Heterogeneity: Not applicable						
Test for overall effect: Z = 1.59 (P = 0.11)						

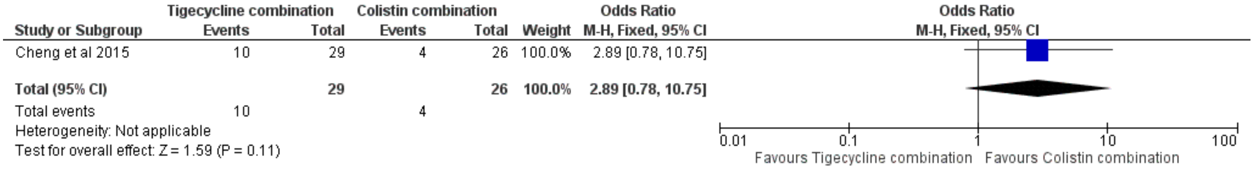

Figure S1.17. Renal impairment: colistin versus tigecycline combinations

	Tigecycline combination		Colistin combination			Odds Ratio
Study or Subgroup	Events	Total	Events	Total	Weight	M-H, Fixed, 95% CI
Cheng et al 2015	5	19	6	16	33.6%	0.60 [0.14, 2.51]
Kim et al 2016	0	30	8	40	50.3%	0.06 [0.00, 1.13]
Seok et al 2021	1	5	34	113	16.1%	0.58 [0.06, 5.39]
**Total (95% CI)**		**54**		**169**	**100.0%**	**0.32 [0.11, 0.92]**
Total events	6		48			

Heterogeneity: Chi² = 2.18, df = 2 (P = 0.34); I² = 8%
Test for overall effect: Z = 2.11 (P = 0.03)

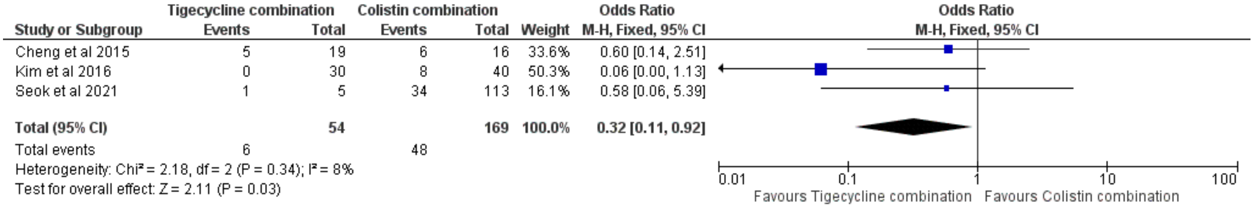

Figure S1.18. Liver enzymes abnormalities: colistin versus tigecycline combinations

	Tigecycline combination		Colistin combination			Odds Ratio
Study or Subgroup	Events	Total	Events	Total	Weight	M-H, Fixed, 95% CI
Seok et al 2021	1	5	16	113	100.0%	1.52 [0.16, 14.44]
**Total (95% CI)**		**5**		**113**	**100.0%**	**1.52 [0.16, 14.44]**
Total events	1		16			

Heterogeneity: Not applicable
Test for overall effect: Z = 0.36 (P = 0.72)

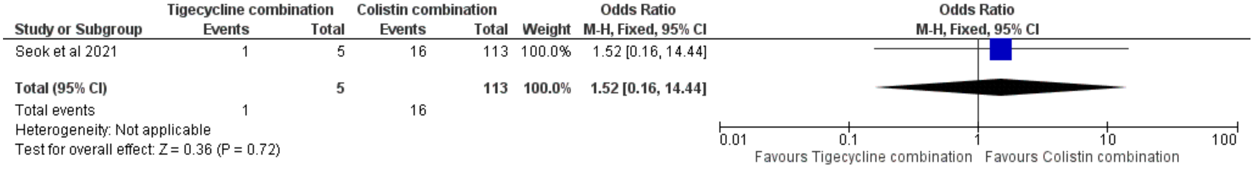

Supplementary Materials S2. Subgroup analysis results

Figure S2.1. Subgroup analysis for clinical cure: colistin versus tigecycline monotherapies

	Tigecycline monotherapy		Colistin monotherapy			Odds Ratio
Study or Subgroup	Events	Total	Events	Total	Weight	M-H, Fixed, 95% CI
Kwon et al 2014	7	16	19	39	41.5%	0.82 [0.25, 2.64]
Oliveira et al 2020	12	31	15	34	58.5%	0.80 [0.30, 2.15]
Seok et al 2021	5	7	28	56	0.0%	2.50 [0.45, 13.98]
**Total (95% CI)**		**47**		**73**	**100.0%**	**0.81 [0.38, 1.72]**
Total events	19		34			
Heterogeneity: Chi² = 0.00, df = 1 (P = 0.98); I² = 0%						
Test for overall effect: Z = 0.55 (P = 0.58)						

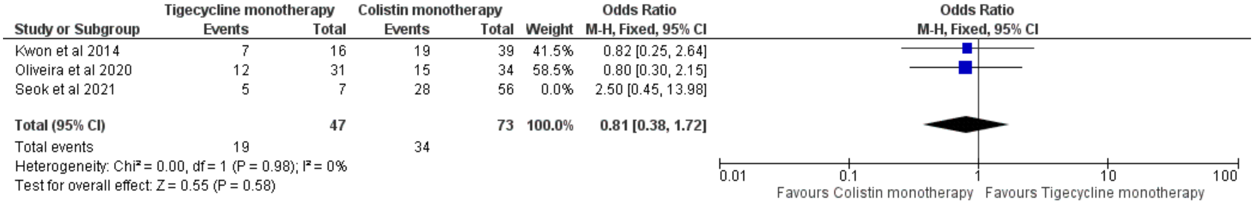

Figure S2.2. Subgroup analysis for 28-day and 30-day mortalities: colistin versus tigecycline monotherapies

	Tigecycline monotherapy		Colistin monotherapy			Odds Ratio
Study or Subgroup	Events	Total	Events	Total	Weight	M-H, Fixed, 95% CI
Diakos et al 2014	11	27	12	22	57.8%	0.57 [0.18, 1.79]
Ku et al 2012	0	16	30	71	0.0%	0.04 [0.00, 0.71]
Lopez-Cortes et al 2014	1	5	12	46	13.9%	0.71 [0.07, 6.98]
Papadimitriou-Olivgeris et al 2014	4	11	1	2	7.9%	0.57 [0.03, 11.85]
Seok et al 2021	2	5	28	56	20.3%	0.67 [0.10, 4.30]
**Total (95% CI)**		**48**		**126**	**100.0%**	**0.61 [0.26, 1.44]**
Total events	18		53			
Heterogeneity: Chi² = 0.04, df = 3 (P = 1.00); I² = 0%						
Test for overall effect: Z = 1.12 (P = 0.26)						

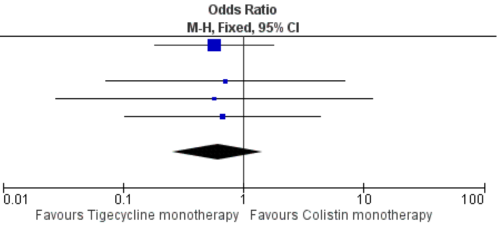

Figure S2.3. Subgroup analysis for in-hospital mortality, excluding ICU and infection-caused mortality: colistin versus tigecycline monotherapies

	Tigecycline monotherapy		Colistin monotherapy			Odds Ratio
Study or Subgroup	Events	Total	Events	Total	Weight	M-H, Random, 95% CI
Ku et al 2012	0	16	26	71	15.1%	0.05 [0.00, 0.90]
Kwon et al 2014	9	16	17	39	34.0%	1.66 [0.51, 5.38]
Liang et al 2017	37	84	6	34	0.0%	3.67 [1.38, 9.80]
Oliveira et al 2020	3	31	2	34	24.6%	1.71 [0.27, 11.01]
Seok et al 2021	5	7	28	56	26.3%	2.50 [0.45, 13.98]
Zarkotou et al 2011	2	5	4	7	0.0%	0.50 [0.05, 5.15]
**Total (95% CI)**		**70**		**200**	**100.0%**	**1.10 [0.28, 4.30]**
Total events	17		73			
Heterogeneity: Tau² = 1.06; Chi² = 6.96, df = 3 (P = 0.07); I² = 57%						
Test for overall effect: Z = 0.14 (P = 0.89)						

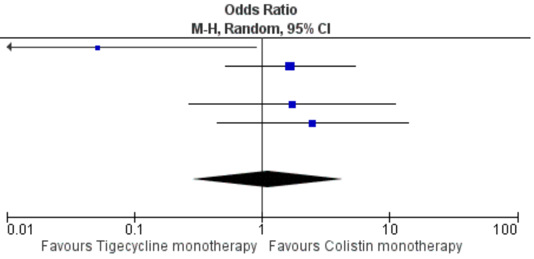

Figure S2.4. Subgroup analysis for nephrotoxicity: colistin versus tigecycline monotherapies

	Tigecycline monotherapy		Colistin monotherapy			Odds Ratio
Study or Subgroup	Events	Total	Events	Total	Weight	M-H, Random, 95% CI
Chuang et al 2014	6	175	12	119	40.9%	0.32 [0.12, 0.87]
Kwon et al 2014	2	16	17	39	35.4%	0.18 [0.04, 0.93]
Oliveira et al 2020	7	31	20	34	0.0%	0.20 [0.07, 0.60]
Seok et al 2021	7	7	26	58	23.7%	18.40 [1.00, 337.13]
**Total (95% CI)**		**198**		**216**	**100.0%**	**0.69 [0.09, 5.33]**
Total events	15		55			
Heterogeneity: Tau² = 2.41; Chi² = 8.74, df = 2 (P = 0.01); I² = 77%						
Test for overall effect: Z = 0.36 (P = 0.72)						

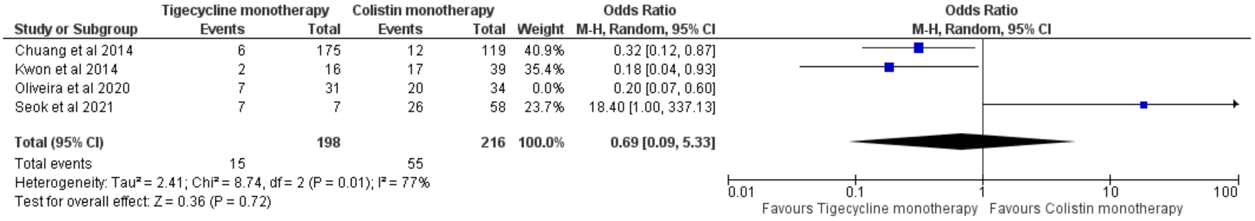

Figure S2.5. Subgroup analysis for 30-day mortality with the colistin monotherapy only: monotherapy versus combination

Study or Subgroup	Monotherapy Events	Monotherapy Total	Combination therapy Events	Combination therapy Total	Weight	Odds Ratio M-H, Fixed, 95% CI
Amat et al 2018	47	76	24	42	47.5%	1.22 [0.56, 2.62]
Chusri et al 2019	5	14	4	14	0.0%	1.39 [0.28, 6.84]
Diakos et al 2014	11	27	5	21	0.0%	2.20 [0.62, 7.79]
Diakos et al 2014	10	22	5	21	11.2%	2.67 [0.72, 9.87]
Ku et al 2012	0	16	8	19	0.0%	0.04 [0.00, 0.78]
Ku et al 2012	30	71	8	19	29.3%	1.01 [0.36, 2.80]
Lopez-Cortes et al 2014	12	46	2	9	10.0%	1.24 [0.22, 6.79]
Lopez-Cortes et al 2014	1	5	2	9	0.0%	0.88 [0.06, 12.97]
Papadimitriou-Olivgeris et al 2014	1	2	1	2	2.0%	1.00 [0.02, 50.40]
Papadimitriou-Olivgeris et al 2014	4	11	1	2	0.0%	0.20 [0.01, 6.04]
**Total (95% CI)**		**217**		**93**	**100.0%**	**1.31 [0.78, 2.21]**
Total events	100		40			

Heterogeneity: Chi² = 1.45, df = 4 (P = 0.84); I² = 0%
Test for overall effect: Z = 1.03 (P = 0.30)

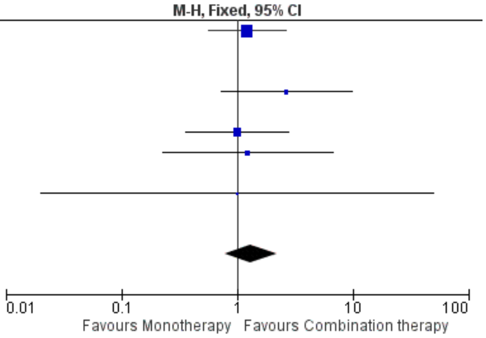

Figure S2.6. Subgroup analysis for 30-day mortality with the tigecycline monotherapy only: monotherapy versus combination

Study or Subgroup	Monotherapy Events	Monotherapy Total	Combination therapy Events	Combination therapy Total	Weight	Odds Ratio M-H, Fixed, 95% CI
Amat et al 2018	47	76	24	42	0.0%	1.22 [0.56, 2.62]
Chusri et al 2019	5	14	4	14	16.4%	1.39 [0.28, 6.84]
Diakos et al 2014	11	27	5	21	21.2%	2.20 [0.62, 7.79]
Diakos et al 2014	10	22	5	21	0.0%	2.67 [0.72, 9.87]
Ku et al 2012	0	16	8	19	48.3%	0.04 [0.00, 0.78]
Ku et al 2012	30	71	8	19	0.0%	1.01 [0.36, 2.80]
Lopez-Cortes et al 2014	12	46	2	9	0.0%	1.24 [0.22, 6.79]
Lopez-Cortes et al 2014	1	5	2	9	7.3%	0.88 [0.06, 12.97]
Papadimitriou-Olivgeris et al 2014	1	2	1	2	0.0%	1.00 [0.02, 50.40]
Papadimitriou-Olivgeris et al 2014	4	11	1	2	6.9%	0.57 [0.03, 11.85]
**Total (95% CI)**		**73**		**65**	**100.0%**	**0.82 [0.39, 1.71]**
Total events	21		20			

Heterogeneity: Chi² = 6.79, df = 4 (P = 0.15); I² = 41%
Test for overall effect: Z = 0.54 (P = 0.59)

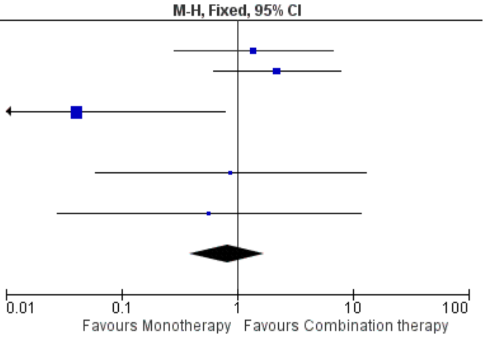

Figure S2.7. Subgroup analysis for 30-day mortality, excluding 3-month mortality: monotherapy versus combination

Study or Subgroup	Monotherapy Events	Monotherapy Total	Combination therapy Events	Combination therapy Total	Weight	Odds Ratio M-H, Fixed, 95% CI
Amat et al 2018	47	76	24	42	44.3%	1.22 [0.56, 2.62]
Chusri et al 2019	5	14	4	14	9.7%	1.39 [0.28, 6.84]
Diakos et al 2014	11	27	5	21	12.5%	2.20 [0.62, 7.79]
Diakos et al 2014	10	22	5	21	10.5%	2.67 [0.72, 9.87]
Ku et al 2012	0	16	8	19	0.0%	0.04 [0.00, 0.78]
Ku et al 2012	30	71	8	19	0.0%	1.01 [0.36, 2.80]
Lopez-Cortes et al 2014	12	46	2	9	9.3%	1.24 [0.22, 6.79]
Lopez-Cortes et al 2014	1	5	2	9	4.3%	0.88 [0.06, 12.97]
Papadimitriou-Olivgeris et al 2014	1	2	1	1	3.4%	0.33 [0.01, 16.80]
Papadimitriou-Olivgeris et al 2014	4	11	1	1	6.0%	0.20 [0.01, 6.04]
**Total (95% CI)**		**203**		**118**	**100.0%**	**1.40 [0.86, 2.30]**
Total events	91		44			

Heterogeneity: Chi² = 3.46, df = 7 (P = 0.84); I² = 0%
Test for overall effect: Z = 1.34 (P = 0.18)

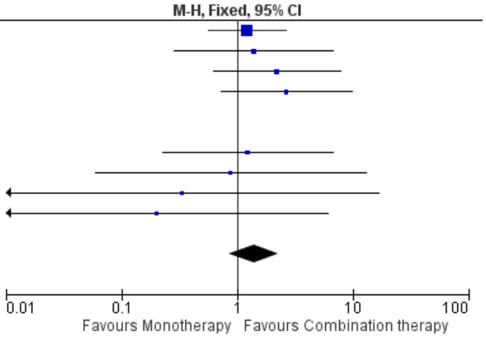

Figure S2.8. Subgroup analysis for in-hospital mortality with the colistin monotherapy only: monotherapy versus combination

Study or Subgroup	Monotherapy Events	Monotherapy Total	Combination therapy Events	Combination therapy Total	Weight	Odds Ratio M-H, Random, 95% CI
Chusri et al 2019	6	14	5	14	0.0%	1.35 [0.29, 6.18]
Ku et al 2012	26	71	7	19	60.9%	0.99 [0.35, 2.83]
Ku et al 2012	0	16	8	19	0.0%	0.04 [0.00, 0.78]
Zarkotou et al 2011	2	5	0	9	0.0%	13.57 [0.51, 358.64]
Zarkotou et al 2011	4	7	0	9	39.1%	24.43 [1.03, 580.63]
**Total (95% CI)**		**78**		**28**	**100.0%**	**3.46 [0.15, 78.21]**
Total events	30		7			

Heterogeneity: Tau² = 3.86; Chi² = 3.66, df = 1 (P = 0.06); I² = 73%
Test for overall effect: Z = 0.78 (P = 0.43)

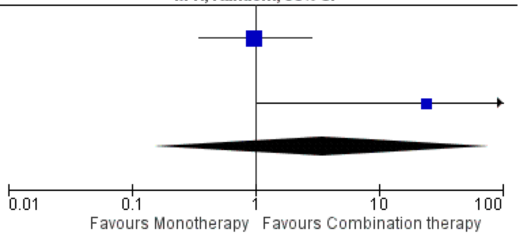

Figure S2.9. Subgroup analysis for in-hospital mortality with the tigecycline monotherapy only: monotherapy versus combination

Study or Subgroup	Monotherapy Events	Monotherapy Total	Combination therapy Events	Combination therapy Total	Weight	Odds Ratio M-H, Random, 95% CI
Chusri et al 2019	6	14	5	14	40.7%	1.35 [0.29, 6.18]
Ku et al 2012	26	71	7	19	0.0%	0.99 [0.35, 2.83]
Ku et al 2012	0	16	8	19	30.8%	0.04 [0.00, 0.78]
Zarkotou et al 2011	2	5	0	9	28.6%	13.57 [0.51, 358.64]
Zarkotou et al 2011	4	7	0	9	0.0%	24.43 [1.03, 580.63]
**Total (95% CI)**		**35**		**42**	**100.0%**	**0.89 [0.05, 15.23]**
Total events	8		13			

Heterogeneity: Tau² = 4.56; Chi² = 7.50, df = 2 (P = 0.02); I² = 73%
Test for overall effect: Z = 0.08 (P = 0.94)

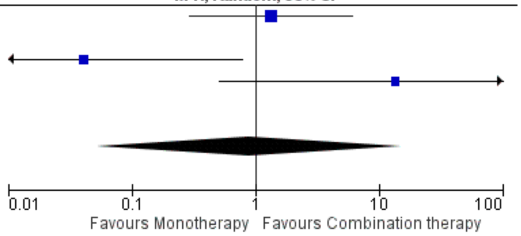

Figure S2.10. Subgroup analysis for in-hospital mortality, excluding infection-caused mortality: monotherapy versus combination

Study or Subgroup	Monotherapy Events	Monotherapy Total	Combination therapy Events	Combination therapy Total	Weight	Odds Ratio M-H, Random, 95% CI
Chusri et al 2019	6	14	5	14	36.6%	1.35 [0.29, 6.18]
Ku et al 2012	26	71	7	19	45.4%	0.99 [0.35, 2.83]
Ku et al 2012	0	16	8	19	18.1%	0.04 [0.00, 0.78]
Zarkotou et al 2011	2	5	0	9	0.0%	13.57 [0.51, 358.64]
Zarkotou et al 2011	4	7	0	9	0.0%	24.43 [1.03, 580.63]
**Total (95% CI)**		**101**		**52**	**100.0%**	**0.62 [0.14, 2.82]**
Total events	32		20			

Heterogeneity: Tau² = 1.02; Chi² = 4.94, df = 2 (P = 0.08); I² = 60%
Test for overall effect: Z = 0.61 (P = 0.54)

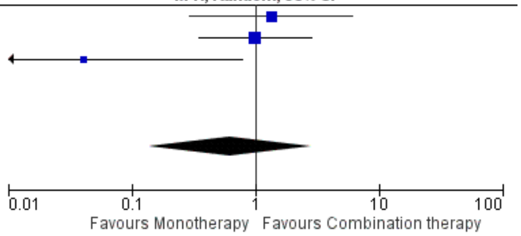

Figure S2.11. Subgroup analysis for in-hospital mortality, excluding ICU mortality: colistin versus tigecycline combinations

Study or Subgroup	Tigecycline combination Events	Tigecycline combination Total	Colistin combination Events	Colistin combination Total	Weight	Odds Ratio M-H, Fixed, 95% CI
Cheng et al 2015	20	29	13	26	33.2%	2.22 [0.74, 6.68]
Kim et al 2016	14	30	16	40	0.0%	1.31 [0.50, 3.42]
Kim et al 2016	15	30	20	40	66.8%	1.00 [0.39, 2.58]
Liang et al 2017	10	32	12	33	0.0%	0.80 [0.28, 2.23]
Zarkotou et al 2011	0	3	0	2		Not estimable
**Total (95% CI)**		**59**		**66**	**100.0%**	**1.41 [0.69, 2.86]**
Total events	35		33			

Heterogeneity: Chi² = 1.16, df = 1 (P = 0.28); I² = 14%
Test for overall effect: Z = 0.94 (P = 0.35)

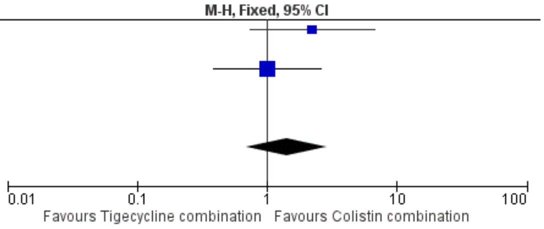

Supplement: Supplementary file 1 [file antibiotics-11-01630-s001.zip › antibiotics-1891757-supplementary.pdf]
